# Are novel and co‐xenic associations common in alien fungal and fungus‐like plant pathogens?

**DOI:** 10.1111/nph.71455

**Published:** 2026-08-24

**Authors:** Anna Schertler, Bernd Lenzner, Stefan Dullinger, Dietmar Moser, Adrián García‐Rodríguez, Irmgard Krisai‐Greilhuber, Hermann Voglmayr, Jennifer L. Bufford, Alberto Santini, Luisa Ghelardini, César Capinha, Luís Reino, Michael J. Wingfield, Marco Thines, Pedro Talhinhas, Wayne Dawson, Mark van Kleunen, Holger Kreft, Jan Pergl, Petr Pyšek, Patrick Weigelt, Marten Winter, Franz Essl

**Affiliations:** ^1^ Division of BioInvasions, Global Change & Macroecology, Department of Botany and Biodiversity Research University of Vienna 1030 Vienna Austria; ^2^ Vienna Doctoral School of Ecology and Evolution University of Vienna 1030 Vienna Austria; ^3^ Division of Biodiversity Dynamics and Conservation, Department of Botany and Biodiversity Research University of Vienna 1030 Vienna Austria; ^4^ Department of Botany and Biodiversity Research University of Vienna 1030 Vienna Austria; ^5^ Manaaki Whenua ‐ Landcare Research Lincoln 7640 New Zealand; ^6^ Italian National Research Council (CNR) Institute for Sustainable Plant Protection 50019 Sesto Fiorentino Italy; ^7^ DAGRI, Department of Agricultural, Food, Environmental and Forest Sciences and Technologies University of Florence 50144 Florence Italy; ^8^ Centro de Estudos Geográficos, Instituto de Geografia e Ordenamento do Território Universidade de Lisboa 1600‐276 Lisbon Portugal; ^9^ Laboratório Associado TERRA 1349‐017 Lisbon Portugal; ^10^ CIBIO, Centro de Investigação em Biodiversidade e Recursos Genéticos, InBIO Laboratório Associado, Campus de Vairão Universidade do Porto 4485‐661 Vairão Portugal; ^11^ Centro de Estudos Florestais, Instituto Superior de Agronomia Universidade de Lisboa 1349‐017 Lisbon Portugal; ^12^ BIOPOLIS Program in Genomics, Biodiversity and Land Planning, CIBIO 4485‐661 Vairão Portugal; ^13^ Forestry and Agricultural Biotechnology Institute (FABI) University of Pretoria Pretoria 0002 South Africa; ^14^ Senckenberg Biodiversity and Climate Research Centre (SBiK‐F) 60325 Frankfurt am Main Germany; ^15^ Department of Biological Sciences, Institute of Ecology, Evolution and Diversity Goethe University Frankfurt am Main 60438 Frankfurt am Main Germany; ^16^ LEAF Research Centre, Instituto Superior de Agronomia Universidade de Lisboa 1349‐017 Lisbon Portugal; ^17^ Department of Evolution, Ecology, and Behaviour, Institute of Infection, Veterinary and Ecological Sciences University of Liverpool Liverpool L69 7ZB UK; ^18^ Ecology, Department of Biology University of Konstanz 78464 Konstanz Germany; ^19^ Zhejiang Key Laboratory for Restoration of Damaged Coastal Ecosystems, School of Life Sciences Taizhou University Taizhou Zhejiang 318000 China; ^20^ Biodiversity, Macroecology & Biogeography University of Göttingen 37077 Göttingen Germany; ^21^ Centre of Biodiversity and Sustainable Land Use (CBL) University of Göttingen 37077 Göttingen Germany; ^22^ Campus‐Institut Data Science, University of Göttingen 37073 Göttingen Germany; ^23^ Institute of Botany, Department of Invasion Ecology Czech Academy of Sciences 252 43 Průhonice Czech Republic; ^24^ Department of Ecology, Faculty of Science Charles University 128 44 Prague Czech Republic; ^25^ Department of Environmental Science, Radboud Institute for Biological and Environmental Sciences (RIBES) Radboud University 6525 AJ Nijmegen the Netherlands; ^26^ iDiv, German Centre for Integrative Biodiversity Research 04103 Leipzig Germany

**Keywords:** biological invasion, co‐xenic novel association, emerging infectious disease, host jump, host switch, interspecific interaction, novel association, parasite

## Abstract

- Impacts of alien fungal and fungus‐like plant pathogens depend on host interactions, including novel associations with native plants and co‐xenic associations with alien plants that do not share the same area of origin, both largely unquantified globally.
- Combining global distribution and association data, we characterised associations across pathogens' introduced regions, calculated host species richness (SR) and phylogenetic divergence, and examined predictors of novel/co‐xenic associations.
- Novel/co‐xenic alien pathogen–plant association records were common (53%) and dominated by widespread generalists. They were found in about one‐third of alien pathogens examined, mostly within their known phylogenetic host range. However, 46% of associations remained unclassified due to limited knowledge of pathogen biogeography. Novel/co‐xenic associations were more likely for pathogens with larger introduced ranges, wider phylogenetic host range, economically used hosts co‐occurring in the region, and host communities phylogenetically similar to the recipient regions' plant community. They were also more likely for host plants with no economic use and restricted native ranges, and in regions with higher plant SR.
- Novel/co‐xenic associations are common and shaped by contact opportunity, phylogenetic host range, and regional community composition. Unravelling fungal and fungus‐like pathogen biogeography is key to assessing their full magnitude.

## Graphical abstract

Possible scenarios for plant–pathogen associations during invasion. An introduced plant may occur in a new area without its pathogen (enemy release) or together with its pathogen (co‐introduction). The presence of the alien plant (in red) and its co‐introduced pathogen (in dark red) can result in pathogen spillover to co‐occurring plants. Host jumps to native plants (in green) result in novel associations, and host jumps to alien plants that do not naturally co‐occur with the pathogen in its native range lead to co‐xenic associations.

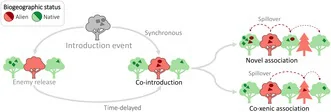

## Introduction

The numerous implications of *biological invasions* (see Table 1, for definitions of italicised terms) have been increasingly recognised, with *invasive alien taxa* being one of the main drivers of ongoing biodiversity loss and causing enormous economic costs world‐wide (IPBES, 2019, 2023; Diagne *et al*., 2021). This is especially true for *alien pathogens* (here defined as fungal and fungus‐like plant pathogens), in which geographical, environmental, and evolutionary barriers limit disease outbreaks (Paap *et al*., 2022). Human‐mediated introductions of pathogens bypass geographical barriers, facilitate *host jumps* (Burgess *et al*., 2016; Thines *et al*., 2023), and potentially lead to emerging infectious diseases (Anderson *et al*., 2004). Alien pathogens encountering naïve hosts can cause high mortality rates and population declines, with cascading effects on community composition and ecosystem functions, for example via increased input of dead material (Vacher *et al*., 2010; Fones *et al*., 2017; Thakur *et al*., 2019). In addition, the spread of pathogens has significant direct negative effects on humans, for example through yield losses in forestry and agriculture, which not only lead to economic losses but also can jeopardise food security (Bebber *et al*., 2014; Fones *et al*., 2017; Case *et al*., 2025).

**Table 1:** Glossary of key terms.

Term	Definition
Alien taxa Established alien taxa Invasive alien taxa	Species or infraspecific taxa introduced beyond their native range (syn. non‐native, introduced, exotic). Some alien taxa *establish* by forming viable populations and a subset becomes *invasive*, causing negative impacts on native biota and ecosystems, including nature's contributions to people (IPBES, 2023). *Note: for this study*, *confirmed alien taxa and those considered likely alien* (*cryptogenic*) *are grouped together*.
Biological invasion	Process of the human‐mediated intentional or unintentional introduction of taxa to new areas beyond their native range, followed by establishment, spread and potential impacts (IPBES, 2023).
Co‐introduction	Associations between an alien plant and an alien pathogen from the plants' native range. These can result from simultaneous introduction, for example through asymptomatically infected plants or plant parts, associated soil or insect vectors, or from time‐delayed reassociation events (Dickie *et al*., 2017).
Cryptogenic	Taxa of unknown biogeographic origin, but likely alien, indicated by factors such as implausible distribution patterns, association with introduction pathways, recording history or species ecology, with some remaining uncertainty (Essl *et al*., 2018). *Note: We use the term strictly in a biogeographic sense; other definitions exist* (*e.g. a disease of unknown cause*).
Host jump Host switch Host range expansion	Colonisation of a new host species by a pathogen happens through host jumps. *Host switches* lead to increasing genetic differentiation and speciation of the pathogen, while *host range expansion* not directly alters the pathogens' gene pool, therefore both terms are distinguished by their long‐term effect (Thines, 2019). *Note: For the sake of simplicity*, *we refer to host jumps throughout this work*.
Inoculum	Infective biological material of a pathogen, such as viable spores or mycelial fragments. Often the likelihood of infection and disease severity is dependent on the quantity of inoculum and duration of exposure (*inoculum pressure*) (Parker *et al*., 2015).
Novelty Novel association Co‐xenic association	Ecological novelty describes systems that have not previously existed in that form and can be applied at a range of organisational scales (Dunn & Hatcher, 2015). Alien taxa can have novel interspecific interactions, through biogeographically *novel associations* (i.e. those between a native plant and an alien pathogen^1^) or *co‐xenic associations* ^2^ (i.e. between an alien plant and an alien pathogen, where both do not naturally co‐occur) (Dickie *et al*., 2017). ^1^ *Note: Novel associations also can occur between a native pathogen and an alien plant*, *but those are not dealt with in this work*. ^2^ *Note: For improved readability throughout the manuscript we choose to use ‘co‐xenic’ as used in* (van Wilgen *et al*., 2022) *instead of ‘co‐xenic novel’* (Dickie *et al*., 2017).
Pathogen (as used in this work)	Fungal and fungus‐like plant pathogens, that is, those members of Fungi and Oomycota that are parasitic or partly parasitic, negatively affect plant fitness and able to cause disease in plant hosts at some point of their life cycle. *Note: Different fungal lifestyles* (*e.g*., *pathogens*, *mutualists*, *endophytes*, *saprobes*) *are not always clear‐cut and disease emergence depends on several factors* (*‘disease triangle’*) (Termorshuizen, 2018). *Note: While this study focusses on Fungi and Oomycota*, *the definition of ‘pathogen’ applies more broadly across potential pathogenic taxa*.
Pathogen spillover	Event, where a pathogen reaches high abundance in a highly competent or abundant host, increasing inoculum pressure, and the likelihood of spillover to another host species, potentially resulting in a host jump. Transmission from the reservoir population drives the epidemics in the recipient host (Power & Mitchell, 2004).

**Fig. 1:**
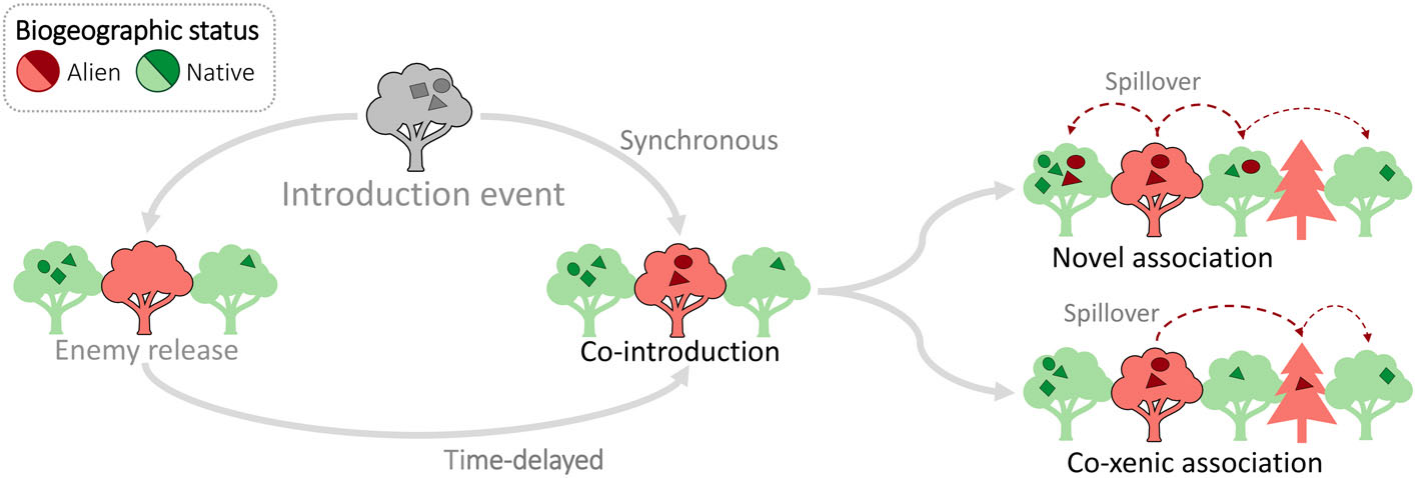
Possible scenarios for plant–pathogen associations during invasion. An introduced plant may occur in a new area without its pathogen (enemy release) or together with its pathogen (co‐introduction). This can happen synchronously, when both are initially introduced together, or through time‐delayed reassociation, when the pathogen follows later. Pathogens may be introduced as contaminants of their hosts, associated soil (e.g. in plant pots), plant parts or animals (e.g. bark beetles) (Hulme *et al*., 2008). The presence of the alien plant (in red) and its co‐introduced pathogen (in dark red) can result in pathogen spillover to co‐occurring plants. Host jumps to native plants (in green) result in novel associations, and host jumps to alien plants that do not naturally co‐occur with the pathogen in its native range lead to co‐xenic associations (Dickie *et al*., 2017).

Importantly, impacts of alien pathogens on humans, biodiversity, and ecosystems ultimately depend on the associations they establish in the introduced range (Burgess *et al*., 2016; Dickie *et al*., 2017). Biotic interactions of alien pathogens can emerge from original host associations or from new associations, in which a previously nonexistent association is formed, resulting in *ecological novelty* (Dunn & Hatcher, 2015; Dickie *et al*., 2017) (Fig. 1). *Co‐introduction* can happen either simultaneously with the introduction of the plant host or time‐delayed, following initial enemy release (i.e. introduction of the plant without its pathogens). Co‐introduced pathogens may form novel associations via *pathogen spillover* onto co‐occurring plants in the introduced range (Power & Mitchell, 2004; Ghelardini *et al*., 2016; Thines, 2019). *Novel associations* can lead to severe impacts on ecosystems, for example by causing major diseases, while *co‐xenic associations* may decrease alien plant invasion success; both can result in economic losses, particularly when economically used plants are affected (Dickie *et al*., 2017). Novel hosts that are competent, namely those that support pathogen reproduction, can act as additional reservoir populations, thereby increasing prevalence, *inoculum pressure*, and the risk of spillover to further alien or native biota (Parker *et al*., 2015). Thus, invasions by alien pathogens potentially influence both alien and native plants. Biogeographically novel associations may have important implications for disease dynamics in recipient communities (Gilbert, 2002; Power & Mitchell, 2004; Newcombe & Dugan, 2010; Dickie *et al*., 2017).

Examples of severe epidemics triggered by novel associations include the chestnut blight (*Cryphonectria parasitica*) on American chestnut (*Castanea dentata*), ash dieback (*Hymenoscyphus fraxineus*) on the European ash (*Fraxinus excelsior*) (Gross *et al*., 2014; Rigling & Prospero, 2018), and Phytophthora dieback (*Phytophthora cinnamomi* and other related species) on many different plant taxa globally (Cahill *et al*., 2008; Burgess & Wingfield, 2017; Shakya *et al*., 2021). Myrtle rust (*Austropuccinia psidii*) jumped from South American Myrtaceae to introduced *Eucalyptus* species, causing extensive economic damage in plantations across South America (Glen *et al*., 2007; Stewart *et al*., 2018; da Silva *et al*., 2026). It has since spread globally, including to Australia, the native range of most *Eucalyptus* species, where it now threatens native Myrtaceae (Carnegie & Pegg, 2018; Stewart *et al*., 2018). Its spread is further promoted by a co‐xenic association with *Syzygium jambos*, the widely cultivated invasive Southeast Asian rose apple (Uchida & Loope, 2009; Soewarto *et al*., 2018). Similarly, *Teratosphaeria destructans*, originating from Southeast Asia, forms co‐xenic associations with eucalypts in planted forest stands in South Africa (Andjic *et al*., 2019). Novel and co‐xenic associations may have facilitated the global spread of these pathogens, by providing new widely planted and economically important reservoir hosts and therefore increased transport opportunities (Burgess & Wingfield, 2017).

Most pathogens have phylogenetically conserved host ranges, reflecting phylogenetic conservatism in host defence and pathogen adaptation (Gilbert & Webb, 2007; Parker *et al*., 2015; Gougherty & Davies, 2021; Lynch *et al*., 2021). Nevertheless, host range can vary considerably (Gougherty, 2023), and for many pathogens, the natural and potential host range breadth is poorly known (Thines *et al*., 2023). Low host specificity can favour invasion success and facilitate the establishment of novel or co‐xenic associations, as seen for broad host range pathogens such as *P. cinnamomi* (Marcot *et al*., 2023). At the same time, highly specialised pathogens can also jump to closely related novel hosts, or successfully spread with globally cultivated host plants (Dickie *et al*., 2017). Host jumps tend to occur among closely related host taxa, but other factors such as geographic distance and a lack of prior contact with the pathogen species or group also influence the likelihood of host jumps (Gilbert & Parker, 2016; Thines, 2019). This is also reflected in the degree of pathogenicity in a host, which seems phylogenetically conserved (Gilbert *et al*., 2015; Lynch *et al*., 2021), but can vary significantly among jumps to closely related hosts with and without co‐evolutionary history (Gougherty & Davies, 2021). Therefore, the phylogenetic composition of the recipient community and its co‐evolutionary history will have implications for the pathogen invasion process and the resulting ecological or economic impacts (Srivastava *et al*., 2012; Gilbert & Parker, 2016).

Regional studies frequently indicate novel associations between plant pathogens and plants, suggesting that these may be relatively common (Desprez‐Loustau *et al*., 2007). In New Zealand, about one‐third of plants and pathogens were found to have at least one biogeographically novel associate, predicted by host taxonomic similarity (Bufford *et al*., 2016). However, it remains unclear how widespread and general these patterns are globally and how they relate to pathogen host range.

In this study, we investigate associations between alien pathogens and seed plants across pathogens' introduced ranges and assess how these associations relate to the pathogens' known host range breadth. To this end, we use several global resources on regional taxon distributions and associations: (a) a global dataset on the distribution and host associations of alien pathogens, (b) the Global Naturalized Alien Flora database (GloNAF; van Kleunen *et al*., 2019; Davis *et al*., 2025), (c) the Global Inventory of Floras and Traits (GIFT; Weigelt *et al*., 2020; Denelle *et al*., 2023), and (d) the World Checklist of Vascular Plants (WCVP; Govaerts *et al*., 2021; Govaerts, 2025). We use these data to address the following questions:

1. How frequent are novel and co‐xenic associations among alien fungal and fungus‐like pathogens and their plant hosts in the introduced range?
2. What is the host range breadth of alien pathogens and how does it change with novelty?
3. What predictors correlate with the probability that an association in the pathogen's introduced range is classified as novel or co‐xenic?

First, we examine the frequency of novel and co‐xenic associations and identify the alien pathogen and plant host taxa contributing most to these patterns. We expect novel and co‐xenic associations to be common globally, consistent with regional evidence (Bufford *et al*., 2016), but probably varying among taxonomic groups.

Next, we characterise pathogen host range breadth by combining available host records globally. We examine basic host specificity (SR, host species richness) and a standardised measure of phylogenetic divergence within host assemblages (ses.MPD; standardised effect size of Mean Pairwise Distance) (Tucker *et al*., 2017). We identify the alien pathogens with the broadest and narrowest host range and show differences between taxonomic groups. We further investigate how phylogenetic divergence changes when considering novel and co‐xenic hosts to identify whether they expand or remain within a pathogen's phylogenetic host range (phylogenetic conservatism).

Finally, we investigate which region‐, pathogen‐, and plant‐related predictors correlate with novelty in the pathogen's alien ranges. We hypothesise that regions with high plant SR provide larger potential host pools. However, the emergence of novel associations will also depend on phylogenetic similarities between the recipient plant community and the pathogen's known host plant species, as well as on co‐evolutionary history (Parker *et al*., 2015; Lynch *et al*., 2021). Pathogens with hosts from species‐rich groups may encounter a larger number of suitable novel hosts. Pronounced human disturbance and intensive food production could lead to higher colonisation pressure and promote spillover events (Bebber *et al*., 2014). Host jumps of alien pathogens co‐occurring on hosts with economic use could be facilitated by increased inoculum pressure (Parker *et al*., 2015). Here, ‘economic use’ follows van Kleunen *et al*. (2020) and includes the uses listed in Diazgranados *et al*. (2020), such as materials, human or animal food, environmental purposes, fuel, and medicine. Pathogens are more likely to be detected in hosts with economic use, as they are usually subject to closer monitoring. Detection may also be higher for woody hosts, which tend to be longer‐lived and provide additional substrate for colonisation (Bufford *et al*., 2016). Globally widespread alien pathogens with broad host ranges have more contact opportunities and possibly genetic preadaptation for host jumps, making them more likely to be involved in novel and co‐xenic associations (Thines, 2019). Similarly, widespread hosts might have increased contact opportunities, but on the other hand, geographically isolated or distant hosts might have lower resistance towards novel pathogens (Thines, 2019).

By quantifying global patterns of novel and co‐xenic pathogen–host associations, this study highlights the importance of species interactions in shaping invasion dynamics. We provide a comparative, quantitative assessment of how frequently such associations occur, how they relate to pathogen host range indices and the region‐, pathogen‐, and host‐level predictors of their occurrence in pathogen's introduced ranges.

## Materials and Methods

The general workflow is shown in Supporting Information Fig. S1, and detailed notes on the workflow are found in the Methods S1.

### Data

#### Taxonomic and spatial scope

We focussed on alien fungal and fungus‐like plant pathogens (see Table 1, for definition), and their seed plant host species (i.e. Spermatophyta). For taxonomic harmonisation, we used a taxonomic backbone following Index Fungorum (RBG Kew, 2021) and MycoBank (Crous *et al*., 2004) for the alien pathogens and WCVP V.14 for plants (Govaerts *et al*., 2021; Govaerts, 2025). We standardised plant names to species level. Pathogen taxa known to be taxonomically problematic, for example due to unclear species boundaries or cryptic species, were excluded from this work (Table S1). Furthermore, we excluded records with unresolved pathogen names, unresolved/missing location, unresolved/missing/vernacular host names, as well as records not resolved to species level.

Distribution and association data were spatially aggregated at the regional level (Fig. S2), using contiguous countries or oceanic islands/archipelagos, based on the Database of Global Administrative Areas (Gadm V.3.6, https://gadm.org/) and the World Geographical Scheme for Recording Plant Distributions (TDWG; Brummitt, 2001). Countries consisting of geographically separated units, such as oceanic islands, were split into separate units (e.g. Spain and the Canary Islands, United States and Hawai’i).

#### Plant species pools

To specify the regional plant species pools and the respective biogeographic status of plant taxa, we combined data from Gift V.3.2 (Weigelt *et al*., 2020; Denelle *et al*., 2023), Glonaf V.2.02 (Davis *et al*., 2025), and Wcvp V.14 (Govaerts *et al*., 2021; Govaerts, 2025). Details on data preparation and cleaning are provided in the Methods S1. GIFT integrates plant distributions from regional checklists and contains information on the species' alien and native status (Weigelt *et al*., 2020). GloNAF is a global database of regional naturalised alien vascular plant distributions (Pyšek *et al*., 2017; van Kleunen *et al*., 2019) and WCVP (Govaerts *et al*., 2021) provides standardised native and alien distribution data at TDWG Level 3 (‘Botanical country’; following the World Geographical Scheme for Recording Plant Distributions; Brummitt, 2001). After taxonomic harmonisation, all plant names were matched to the plant phylogeny of Yang *et al*. (2021), which is based on and extends the dated phylogeny for seed plants published by Smith & Brown (2018).

The final dataset on regional plant species pools contained 1285 830 unique plant–region records across 264 regions and 334 568 plant species (Fig. S3a). Of those records, 13.0% were classified as alien, 0.7% as both, native and alien in a region (e.g. due to taxonomic or spatial standardisation and aggregation), and the remaining 85.8% as native, while 0.5% lack any information on biogeographic status (i.e. unknown status).

#### Pathogen distributions and host associations

We derived information on the distributions and associations of alien pathogens from a comprehensive dataset, which aggregates information on pathogen distribution, hosts and traits and is built upon a wide range of sources including scientific publications, national inventories, alien taxa compendia (e.g. Santini *et al*., 2013; Seebens *et al*., 2017; Pagad *et al*., 2018; CAB International, 2021; Burgess & Scott, 2023), general databases and fungaria not specifically targeting alien taxa (e.g. MyCoPortal (MyCoPortal, 2020), USDA Fungal Databases (Farr & Rossman, 2021), CBS‐KNAW culture collection (Westerdijk Fungal Biodiversity Institute, 2020), and the RBG Kew Fungal Databases HerbIMI and Herbtrack (RBG Kew, 2024a,b); see Table S2, for a list of sources used in the final dataset). The final dataset used for this study is deposited in a public repository (see the Data Availability section). We considered only taxa with information on distribution and hosts in their alien range. The taxonomy and systematics of many fungal groups have experienced profound changes in recent decades (Kruse *et al*., 2018; Talhinhas & Baroncelli, 2021). Therefore, we included a data validation step in which expert feedback and curated datasets for selected taxonomic groups were incorporated for taxonomic, biogeographic, and host‐based corrections. Obviously erroneous host records resulting from misidentifications, changes in taxonomic names over time, and mixing of broad and narrow species concepts were removed (Methods S1). In addition, regional distribution records that were solely supported by such erroneous host records were discarded.

The final dataset contained 1401 pathogen taxa with information on hosts and distribution (965 Ascomycota, 283 Basidiomycota, 3 Blastocladiomycota, 10 Chytridiomycota, and 140 Oomycota). From the collated 34 290 region–pathogen records (Fig. S3b), 31.1% were classified as alien or cryptogenic (i.e. likely alien *sensu* Essl *et al*., 2018), 0.7% as native, and 68.2% as undefined, meaning that explicit information on the biogeographic status was not available or unknown. Around 90% of region–pathogen combinations were supported by at least one record with host information, while *c*. 10% contained only distributional, but no host information.

To estimate the total host range of the alien pathogens, we summarised information on all recorded associations between the pathogen and hosts. This included pathogen–host records in the pathogens' native, alien/cryptogenic (hereafter summarised under ‘alien’), and undefined regions of occurrence as well as host records that were not spatially explicit. While we assume that a recorded presence on a host is most often due to symptomatic plants, we acknowledge that this does not allow inferences about the severity and impact the infection has on the plant host. The resulting dataset of host associations was matched with the plant phylogeny (Yang *et al*., 2021) and contained 42 495 pathogen–host records across 13 630 plant species.

To evaluate the frequency of novel associations in the pathogens' alien range, we classified the biogeographic status of both, pathogen and host, across all spatially explicit pathogen–host records (*n* = 95 060 region–pathogen–host combinations, comprising 12 985 host plant species and 213 standardised regions) (Fig. S3c). To maximise information, the dataset additionally included 1371 supraregional records that were retained when no nested regional records were available, resulting in 96 431 records with spatial information (Table S3). Finally, 1206 pathogen–host combinations lacked standardised spatially explicit distribution information.

**Fig. 2:**
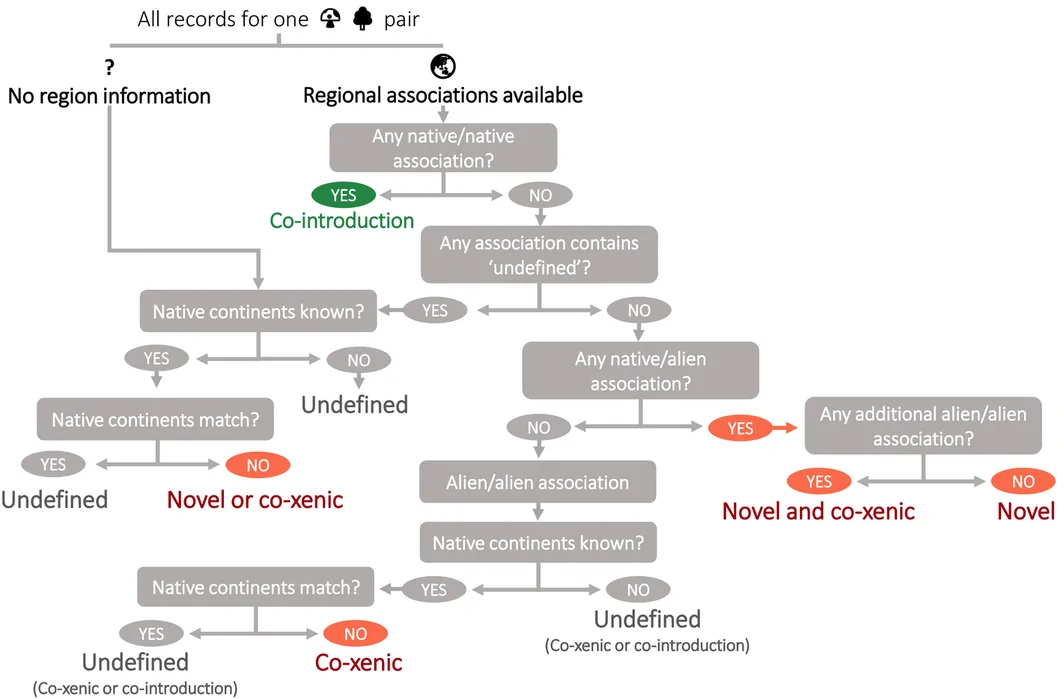
Flowchart for assigning association types to pathogen–host pairs. Co‐introduced associations are those that are known from the native range of the pathogen and plant (native/native). Undefined associations arise when the biogeographic status of at least one taxon (pathogen or host) is undefined/unclear in any regional association record and classification cannot be resolved using native continent information. For pathogen–host pairs in which biogeographic status is unknown, the native continents – if available – are compared: mismatches in native continents indicate that the association is not co‐introduced and thus is a novel or co‐xenic association. Novel associations occur between alien pathogens and native plant hosts. For alien/alien associations, it could be either a co‐xenic or co‐introduced association that lacks records in the native range. To further evaluate this, the native continents are compared: mismatches in native continents indicate co‐xenic associations (i.e. they do not naturally co‐occur in their native ranges), but if the native continents match, then co‐xenic and co‐introduced associations cannot be distinguished. Lastly, through time, it is possible for a pathogen to acquire both novel and co‐xenic associations with the same plant host if the pathogen occurs in the plant's native and introduced ranges. Note that each pathogen–host pair classification is based on all available regional association records. When spatially explicit data are lacking, only native continent comparisons are used. Red endpoints indicate novelty (including novel and/or co‐xenic associations), the green endpoint indicates no novelty and grey endpoints indicate those cases where classification was not possible.

We characterised the type of association by summarising all combinations of biogeographic status for each alien pathogen–host pair into (i) *co‐introduction*; (ii) *undefined* association; and forms of novelty: (iii) *novel* association; (iv) *co‐xenic* association; (v) *novel or co‐xenic* association; or (vi) *novel and co‐xenic* association (i.e. alien pathogen associated with a novel plant host both within and outside of the plant's native range) (Fig. 2). To further resolve undefined associations (including those lacking standardised spatially explicit distribution information), we considered information on native continents of pathogen and plant if available.

To investigate potential correlates of the frequency of novel and co‐xenic associations during pathogen invasions, we filtered the dataset to include only those associations that occur in a pathogen's alien range (*n* = 25 628 region–pathogen–host combinations, comprising 5668 host plant species and 201 regions; Fig. S3d). After restricting to records with complete predictor data, the final dataset used in the model included 24 622 observations (see ‘Correlates of novelty’ in the Analyses section).

### Analyses

#### Frequency of association types

Based on the classification of associations (Fig. 2), we calculated proportions of association types for pathogens recorded in their alien ranges. Fisher's exact tests were used to assess the association between pathogen phylum and class and the frequency of taxa with novel/co‐xenic associations. Classes represented by a single taxon and taxa classified as *Incertae sedis* at class level were excluded from those analyses.

#### Host range breadth of alien pathogens

To characterise the total taxonomic and phylogenetic host range of alien pathogens, we calculated host SR and the standardised effect size of the Mean Pairwise Distance (ses.MPD) following Tucker *et al*. (2017). Phylogenetic diversity (PD) was also evaluated, but excluded from further analyses because it was strongly correlated with SR (Pearson's *r* = 0.93; Fig. S4), which provides a more readily interpretable measure of richness. To calculate MPD, a measure of phylogenetic divergence within host assemblages, at least two host species are required, which was the case for 1248 pathogen taxa (89%). For 153 taxa, only one host species was recorded, and therefore, no phylogenetic divergence indices were calculated. To compare host range with and without novel or co‐xenic associations, we calculated the observed MPD for the full host community of a pathogen (MPD_obs_), and a restricted version (MPD_restr_) excluding hosts identified as novel or co‐xenic, while retaining undefined and co‐introduced hosts. Because native range information is lacking for many pathogens, a direct comparison between native and alien range host community was not possible. This approach therefore provides a conservative proxy for the baseline host community. As MPD is influenced by the plant species pool and host richness, the use of null models to estimate the expected (null) distribution of MPD is recommended when comparing communities of different sizes (Swenson, 2014). For this, we used the pool of plant species potentially available to the alien pathogen in its known global range, that is all plant species recorded in regions across the pathogen's global range. Note that due to the regional scale of this study, we are not able to consider actual co‐occurrences on local scales, and therefore overestimate the potential plant species pool. We used the function ‘mpd’ in the R‐package picante (Kembel *et al*., 2010) and created null models using unconstrained phylogenetic randomisation by shuffling taxon labels in the phylogeny (runs = 999) following Swenson (2014). We then evaluated the degree to which MPD_obs_ and MPD_restr_ deviated from the mean of the null distribution (effect size and quantile position). The quantile position indicates whether the MPD significantly deviates from null with lower and upper tail values indicating clustering and dispersion, respectively. Standardised effect sizes (ses.MPD_obs_, ses.MPD_restr_) were calculated by dividing the effect size by the SD of the null distribution (Fig. S5).

We used Kruskal–Wallis rank sum tests and *post hoc* pairwise comparisons (Wilcoxon test) to test whether SR and ses.MPD differed among classes and, for the restricted indices, between taxa for which novel or co‐xenic associations could be identified in their alien range and those for which this was not the case. Classes represented by a single taxon and taxa classified as *Incertae sedis* were excluded from those analyses. To investigate how phylogenetic divergence of host communities changes when acquiring novel host species, we compared pathogens that extended their phylogenetic host range breadth (ses.MPD_obs_ > ses.MPD_restr_) and those that remained within the already known phylogenetic host range (ses.MPD_obs_ ≤ ses.MPD_restr_).

#### Correlates of novel and co‐xenic associations

To evaluate the relationship of a selection of regional‐, pathogen‐, and plant‐specific predictors (Table 2) with the probability of novelty for an alien region–pathogen–host combination, we fit a binomial generalised linear mixed model (GLMM) by maximum likelihood with a Laplace approximation (Fox *et al*., 2015) using the lme4 package (Bates *et al*., 2015). The binary response variable was whether the region–pathogen–host combination was identified as novel/co‐xenic (novelty = TRUE) or not (co‐introduced/undefined; novelty = FALSE).

**Table 2:** Predictors used to model the probability that an alien region–pathogen–host combination is identified as novel/co‐xenic.

	Predictor	Description	Source	Rationale
Region	Plant species richness (SR_PLANT_)	Number of plant species (log‐transformed)	Plant species pool dataset (GIFT V.3.2, GloNAF V.2.02, WCVP V.14)	Higher plant species richness increases the pool of potential hosts.
	Artificial areas	% landcover	FAO (2013)	Higher levels of human disturbance may increase colonisation pressure, ecosystem invasibility and detection probability, but may reduce the availability of suitable novel hosts.
	Food production	% landcover	Jung *et al*. (2024)	Food production areas may act as pathogen reservoirs and promote spillover, but extensive coverage may reduce the amount of natural ecosystems and thus, availability of suitable novel hosts.
	bmntd_PLANT COM. × HOST COM._	Beta Mean Nearest Taxon Distance between regional plant and host community	Plant species pool dataset (GIFT V.3.2, GloNAF V.2.02, WCVP V.14); Pathogen dataset	Phylogenetically similar plant communities are more likely to contain hosts suitable for colonisation by pathogens.
	Economically used hosts in region	No|yes	Diazgranados *et al*. (2020); van Kleunen *et al*. (2020)	The presence of other economically used hosts in the region may increase propagule pressure through trade and promote spillover, as these plants are often more widespread and abundant.
Pathogen	Host species richness (SR_restr_)	Total number of known hosts excluding those identified as novel/co‐xenic	Pathogen dataset	Pathogens with more known host species are more likely to possess generalist traits that facilitate colonisation of novel host species.
	ses.MPD_restr_	Standardised effect size of Mean Pairwise Distance between hosts, excluding those identified as novel/co‐xenic	Pathogen dataset	Pathogens able to colonise phylogenetically divergent species are more likely to possess generalist traits that facilitate colonisation of novel hosts.
	Alien distribution	Number of regions	Pathogen dataset	Widespread alien pathogens are more successful invaders and also have more contact opportunities with diverse plant taxa around the world.
Plant	Woodiness	No|yes	WCVP V.14	Woody hosts may accumulate more pathogens and have a higher detection probability due to longer lifespans and a greater substrate diversity than herbaceous plants.
	Economic Use	No|yes (all categories)	Diazgranados *et al*. (2020); van Kleunen *et al*. (2020)	Close human association may promote spillover onto native and alien economically used plants, thus creating novel and co‐xenic associations.
	Native distribution	Number of native regions	WCVP V.14	A proxy of endemism. Geographic barriers may have previously played an important role in preventing possible associations and might limit resistance due to shared co‐evolutionary history.
	Number of species in family	Number of plant species in the family	WCVP V.14	Plants that are members of species‐rich families or genera are more prone to experience novel associations, as the pool of pathogens adapted to related species might be larger.
	Number of species in genus	Number of plant species in the genus	WCVP V.14	

This includes predictor name, data type, source, and rationale for inclusion. For categorical predictors, the reference level is underlined. Two predictors are calculated at the region–pathogen scale: bmntd_PLANT COM.×HOST COM._ (Beta Mean Nearest Taxon Distance), and presence of economically used hosts in region. Further details on the plant species pool dataset and the pathogen dataset are provided in the ‘Plant species pools’ and ‘Pathogen distributions and host associations’ subsections in the Materials and Methods section.

We included intercept‐only crossed random effects for region identity, host plant genus, and pathogen genus to account for group effects such as uneven sampling across regions and group‐specific differences among pathogens and plants. Furthermore, we weighted the model by the standardised geometric inverse frequencies of regions, pathogen, and plant taxa (cube root of the product of the inverse‐frequency weights) in the dataset, with the composite weights capped at the 99.5th percentile. This assigns smaller weights to well‐represented regions, such as New Zealand or the United States, common pathogen taxa, such as *P. cinnamomi* Rands, and agricultural plants, such as *Solanum lycopersicum* L.

To evaluate potential predictors of novelty, we investigated the region‐, pathogen‐, and plant‐specific information listed in Table 2. We assessed multicollinearity using variance inflation factors (VIF) and pairwise Pearson correlations. All predictors had a VIF < 2 and pairwise correlations |r| < 0.6, indicating no problematic collinearity (Fig. S6). Continuous predictors were scaled. The model was fitted on 24 622 observations with complete predictor data, representing 1191 pathogen taxa and 5553 plant species, across 201 regions, 375 pathogen genera and 1630 plant genera.

The model was checked for singularity and the significance of the random effects was tested by comparing the fitted model to a reduced model without random effects using a Chi^2^‐test (Chi^2^ = 10553.7, df = 3, *P* < 0.0001). Marginal and conditional pseudo‐*R* ^2^ (*R* ^2^_GLMM_) were computed with the package performance (Lüdecke *et al*., 2021), with the former representing variance explained by fixed effects and the latter the variance explained by the whole model, including random effects (Nakagawa *et al*., 2017). We performed sensitivity analyses to evaluate robustness of model estimates to (1) weighting schemes and random‐effects structure and (2) influential pathogen taxa. For (1), we compared a set of alternative models varying in weighting (uncapped, capped at the 99.9% and 99% quantiles, no weights) and random‐effect hierarchy (genus vs higher taxonomic level) (Fig. S7). For (2), we refitted the final model after excluding the top 1% and 2.5% most influential pathogen taxa based on sampling frequency (Fig. S8). Further details on weighting and sensitivity analyses are provided in the Methods S1.

All analyses were conducted in R v. 4.3.3 (R Core Team, 2023) running in RStudio v. 2025.5.0.496 (Posit team, 2025) using the following packages: ape v. 5.8 (Paradis & Schliep, 2019), bigmemory v. 4.6.4 (Kane *et al*., 2013), corrplot v. 0.92 (Wei & Simko, 2017), data.table v. 1.17.0 (Barrett *et al*., 2025), dbi v. 1.2.2 (R Special Interest Group on Databases (R‐SIG‐DB) *et al*., 2024), gift v. 1.3.2 (Denelle & Weigelt, 2024), lme4 v. 1.1.35.2 (Bates *et al*., 2015), performance v. 0.11.0 (Lüdecke *et al*., 2021), picante v. 1.8.2 (Kembel *et al*., 2010), rsqlite v. 2.3.6 (Müller *et al*., 2024), rwcvp v. 1.2.4 (Brown *et al*., 2023), rwcvpdata v. 0.6.0 (Govaerts *et al*., 2021; Govaerts, 2025), sf v. 1.0.16 (Pebesma, 2018), sjplot v. 2.8.15 (Lüdecke, 2023), terra v. 1.7.71 (Hijmans, 2024), tidyverse v. 2.0.0 (Wickham *et al*., 2019), vegan v. 2.6.4 (Oksanen *et al*., 2022), and viridis v. 0.6.5 (Garnier *et al*., 2024).

## Results

### Frequency of novel and co‐xenic associations

**Fig. 3:**
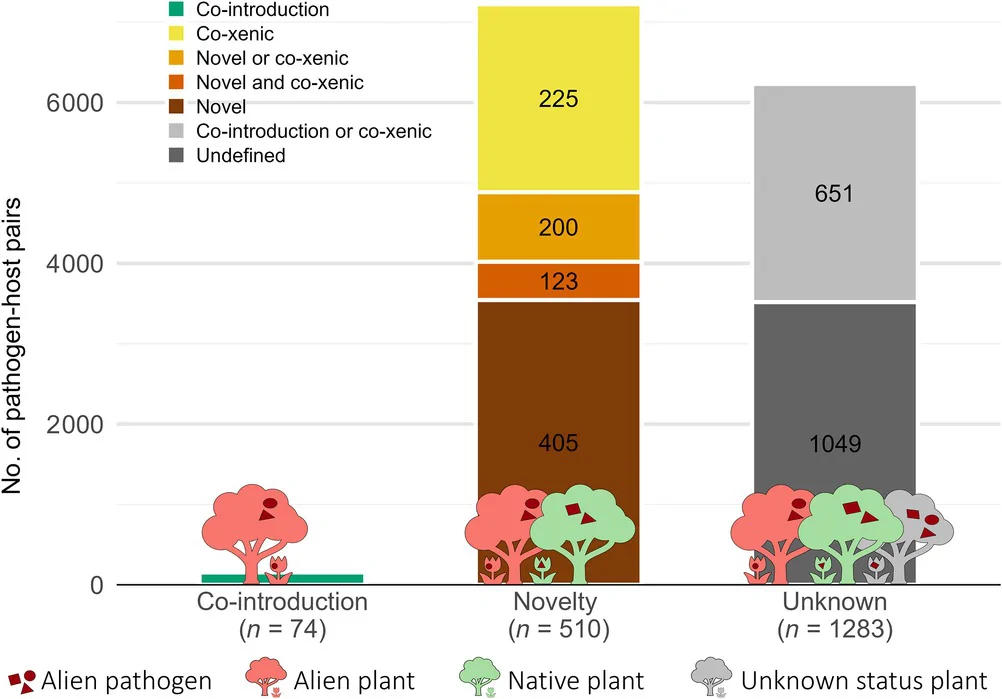
Pathogen–host association types in the pathogen's alien range. Barplot showing frequencies of association types (*n* = 13 609 associations) grouped by novelty. Co‐introduced associations are depicted in green (154 pathogen–host pairs, representing associations of 74 pathogen taxa). Novel associations and co‐xenic associations are depicted in yellow, orange and brown (7223 associations, representing 510 pathogen taxa). Unknown associations are depicted in grey (6232 associations, representing 1283 pathogen taxa). Where a category contains multiple association types, the number inside bars indicates the number of pathogen taxa represented for each type. Note that pathogen taxa with multiple recorded host plants can be represented in multiple association types. Co‐introduced = known from the native range of the pathogen and plant; Novel = alien pathogen on a native host; Co‐xenic = alien pathogen on an alien host with non‐overlapping native ranges; Unknown = status cannot be determined from available records.

Of the association types found in the pathogens' alien ranges (*n* = 13 609 of 42 495 pathogen–host combinations), 53.1% (*n* = 7223) were classified as novel and/or co‐xenic. Notably, nearly half of the associations were of unknown nature (45.8%; *n* = 6232), and only 1.1% (*n* = 154) could be identified as co‐introduction (Fig. 3, Table S4).

Overall, 510 taxa (36.4%) showed novel or co‐xenic associations in their alien range, ranging from 1 to 851 plant species per taxon (mean = 14). This suggests that a few highly generalist species strongly influence the overall frequency of association types. The five species with the highest numbers of novel or co‐xenic associations were as follows: *P. cinnamomi* (851 plant species; 96% of all alien range associations were classified as novel/co‐xenic), *Phytophthora nicotianae* (437, 85%), *Botryosphaeria dothidea* (325, 89%), *A. psidii* (237, 94%), and *Phytophthora palmivora* (191, 87%). Together, these five species accounted for 28.3% of all novel or co‐xenic pathogen–host associations depicted in Fig. 3.

There was a significant association between novel/co‐xenic associations and the five phyla (*n* = 1401) and with the 14 classes, excluding those taxa classified as *Incertae sedis* (*n* = 1389; Fisher's exact test with simulated *P*‐values for phylum: *P* = 0.0011; and class: *P* < 0.0001; Fig. S9). Pairwise comparisons with Bonferroni‐correction indicated that most significant differences were between classes belonging to different phyla: Agaricomyoccetes (Basidiomycota) had a higher proportion of novel/co‐xenic associations compared to Dothideomycetes (*P*‐adj. < 0.0001), Leotiomycetes (*P*‐adj. = 0.0004), Sordariomycetes (*P*‐adj. < 0.0001), Taphrinomycetes (*P*‐adj. = 0.0151) (all Ascomycota), and Peronosporomycetes (Oomycota; *P*‐adj. = 0.0043). Agaricomycetes also showed higher proportions than Exobasidiomycetes (*P*‐adj. = 0.0001), Microbotryomycetes (*P*‐adj. = 0.0028), Pucciniomycetes (*P*‐adj. = 0.0001), Ustilaginomycetes (*P*‐adj. = 0.0015) (all Basidiomycota), and Chytridiomycetes (Chytridiomycota, *P*‐adj. = 0.0380). Dothideomycetes (Ascomycota) had a smaller proportion of novel/co‐xenic associations compared to Leotiomycetes (Ascomycota; *P*‐adj. = 0.0125) and Peronosporomycetes (Oomycota; *P*‐adj. = 0.0012). Differences involving classes with low total sample sizes (e.g. Agaricomycetes *n* = 20, Taphrinomycetes *n* = 11, Chytridiomycetes *n* = 10, and Microbotryomycetes *n* = 6), should be interpreted with caution. This also applies to class‐wise proportions of taxa with novel/co‐xenic associations, in which Agaricomycetes showed the highest proportion (95%, 19/20), followed by Saprolegniomycetes (62.5%, 5/8) and Peronosporomycetes (47.7%, 63/132), whereas Dothideomycetes (27.2%, 132/485) and Sordariomycetes (38.3%, 98/256), exhibited lower values.

The most represented plant families in the pathogens' alien ranges were Myrtaceae (396 plant species with novel/co‐xenic associations; 44% with economic use), Fabaceae (353; 71%), Poaceae (271; 71%), Asteraceae (269; 47%), Proteaceae (214; 29%), and Rosaceae (200; 74%). While Fabaceae and Asteraceae are large families, the strong representation of Myrtaceae, Proteaceae, and Rosaceae, which harbour far fewer species, is noteworthy. In particular, the Proteaceae, with less than 2000 species globally (Govaerts, 2025) and a relatively low proportion of economically used species, stand out, largely due to novel associations. Across all plant host species recorded in the pathogens' alien ranges, the percentage of economically used species was high across families (Mean = 80%; Fig. S10).

The plant species with the highest counts of novel/co‐xenic pathogens in our dataset were mostly widely cultivated species, including fruit trees such as *Citrus* (*C. × aurantium*, bitter orange; 38 pathogen taxa; and *C. × limon*, lemon; 32), *Malus domestica* (apple; 18), *Persea americana* (avocado; 15), and *Prunus* species (*P. persica*, peach; *P. avium*, wild cherry; *P. domestica*, European plum; all 14), and forest trees such as *Quercus* (*Q. ilex*, holm oak; 15; *Q. robur*, pedunculate oak; 14) and *Picea abies* (Norway spruce; 14). By contrast, novel associations only were most frequent in forest and ornamental trees and shrubs of the Northern Hemisphere, including *Quercus robur* (14) and other European and North American congeners, *Fraxinus excelsior* (European ash; 9), *Ulmus glabra* (Scots elm; 9), *Picea abies* (9), *Alnus* *glutinosa* (black alder; 8), *Cornus* *florida* (flowering dogwood; 8), and *Arbutus unedo* (strawberry tree; 8). High counts of novel pathogens were also recorded in range‐restricted woody species of the Southern Hemisphere, including *Agathis australis* (kauri; 10), *Eucalyptus marginata* (jarrah; 8), *Nothofagus fusca* (red southern beech; 7), and *Chamelaucium uncinatum* (Geraldton waxflower; 7). When all association types in the alien ranges of pathogens were considered, the highest numbers of taxa were recorded for widely cultivated crops and plantation species, including *Malus domestica* and *Pinus radiata* (Monterey pine; both 71 recorded alien pathogen taxa), followed by *Vitis vinifera* (grape vine; 53), *Solanum lycopersicum* (tomato), *Lolium perenne* (perennial ryegrass), *Pyrus communis* (pear; all 45), *Triticum* *aestivum* (wheat; 44), *Prunus persica* (43), and *Prunus avium* (42) (Table S5).

### Host range breadth of alien pathogens

**Fig. 4:**
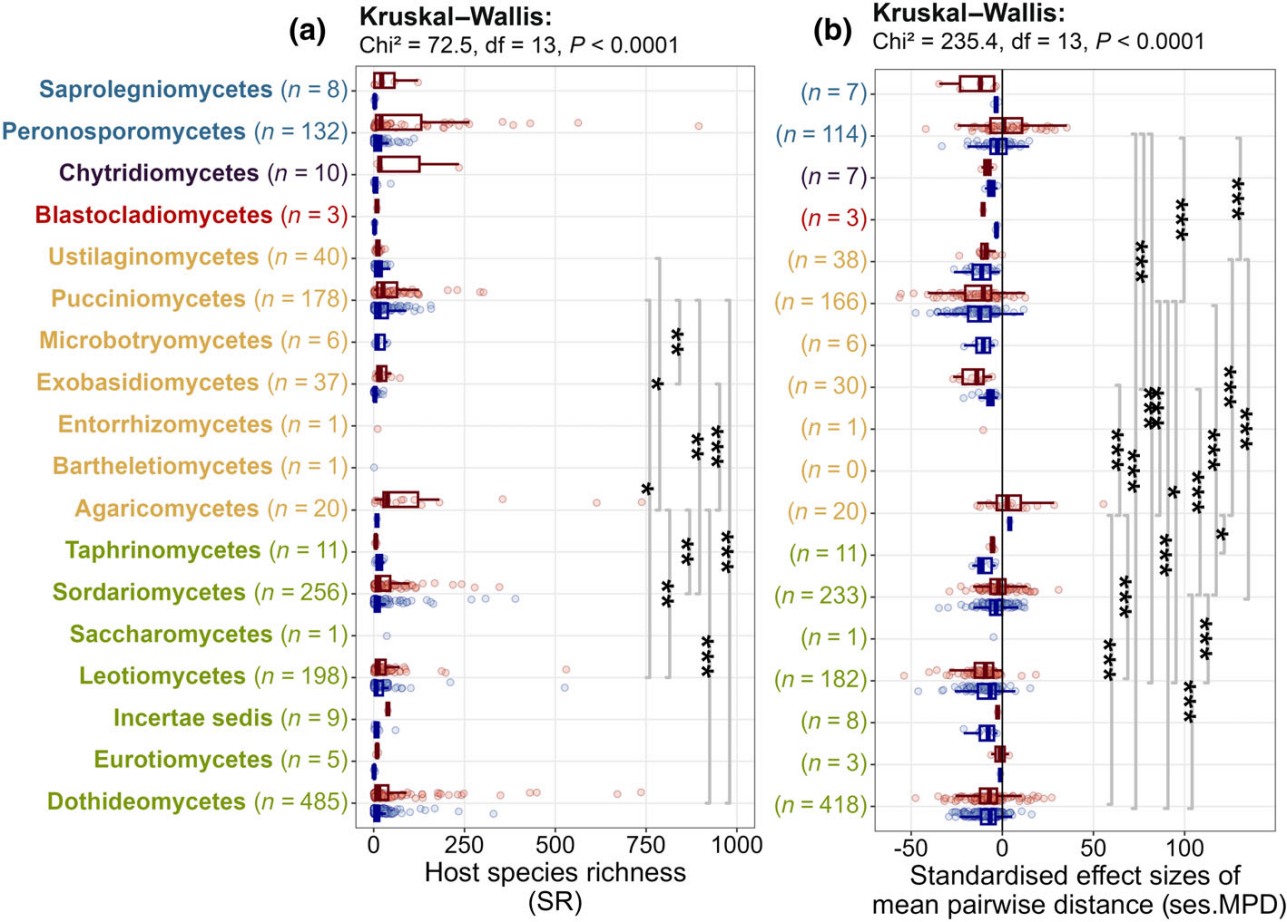
Phylogenetic indices of observed host range breadth by class. Boxplots of (a) observed host species richness (SR_obs_; *n* = 1401 pathogen taxa) and (b) observed host phylogenetic divergence ses.MPD_obs_ (standardised effect size of Mean Pairwise Distance; *n* = 1248 pathogen taxa) are depicted for 17 classes across five phyla, with taxa grouped into those with identified novel or co‐xenic associations (red) vs those with none (blue). Class names are coloured after phylum: green = Ascomycota; yellow = Basidiomycota; red = Blastocladiomycota; dark purple = Chytridiomycota; blue = Oomycota. Boxplots show median (centre line), interquartile range (box), whiskers extending to the most extreme values within 1.5× IQR and points as outliers. The boxplots are accompanied by statistical test results on group differences between classes (Wilcoxon *post hoc* pairwise comparison). Grey bars and the associated asterisks indicate significant pairwise group differences (*, *P* < 0.05; **, *P* < 0.01; ***, *P* < 0.001). Overall difference is indicated by results of a Kruskal–Wallis rank sum test and shown above the panels. Classes represented by a single taxon are shown in the figure but were excluded from statistical analyses. *Incertae sedis* taxa are pooled and shown for completeness but were excluded from statistical analyses due to their ambiguous taxonomic placement.

The number of documented host species (SR) is highly skewed, with only a few alien pathogens showing high numbers of host species (mean = 30, median = 9, max = 895). SR differed significantly within and among Ascomycota and Basidiomycota (Kruskal–Wallis for class‐wise differences in SR_obs_: *n* = 1389, Chi^2^ = 72.5, df = 13, *P* < 0.0001; and SR_restr_: *n* = 1238, Chi^2^ = 56.0, df = 13, *P* < 0.0001), with Wilcoxon *post hoc* pairwise comparisons identifying significant pairwise differences (Figs 4a, S11a). We did not find that, across classes, a higher SR_restr_ was associated with novelty, comparing taxa that have recorded novel/co‐xenic associations vs those that do not (Kruskal–Wallis; *n* = 1238, Chi^2^ = 0.2, df = 1, *P* = 0.63; Fig. S12a). However, when comparing taxa within individual classes, in two classes, the Ustilaginomycetes (Basidiomycota; *n* = 38, Chi^2^ = 7.9, df = 1, *P* = 0.0049) and Taphrinomycetes (Ascomycota; *n* = 11, Chi^2^ = 4.2, df = 1, *P* = 0.04), the opposite trend was observed, that is a lower SR_restr_ was associated with novelty. Importantly, for the latter group, the sample sizes were small and the results were marginally significant (Fig. S12a).

Out of 1248 pathogens with at least two recorded hosts, ses.MPD_obs_ differed significantly from the null model for 1117 taxa and was on average negative (mean = −7.3, min = −67.1, max = 55.5), indicating an overall trend to phylogenetic clustering in host communities (Fig. 4b). While significant phylogenetic clustering was found in ses.MPD_obs_ for 993 taxa across all classes, 124 taxa showed significantly higher phylogenetic divergence than expected (in Basidiomycota: Agaricomycetes, Pucciniomycetes; in Ascomycota: Dothideomycetes, Leotiomycetes, Sordariomycetes; in Oomycota: Peronosporomycetes). The percentage of significantly dispersed phylogenetic host ranges was especially high in Agaricomycetes (63%; 10 taxa) and Peronosporomycetes (41%; 37 taxa) (Table S6).

Classes' ses.MPD differed significantly within and among Ascomycota, Basidiomycota, and Oomycota (Kruskal–Wallis for ses.MPD_obs_: *n* = 1238, Chi^2^ = 235.4, df = 13, *P* < 0.0001; and ses.MPD_restr_: *n* = 1181, Chi^2^ = 222.5, df = 13, *P* < 0.0001 respectively; Wilcoxon *post hoc* pairwise comparison; Figs 4b, S11b). Across classes, a higher ses.MPD_restr_ was associated with novelty (Chi^2^ = 15.3, df = 1, *P* < 0.0001). However, when comparing taxa within individual classes, a significant association between novelty and a higher ses.MPD_restr_ was found only in Peronosporomycetes (Oomycota; *n* = 110, Chi^2^ = 4.3, df = 1, *P* = 0.039) and Ustilaginomycetes (Basidiomycota; *n* = 36, Chi^2^ = 6.52, df = 1, *P* = 0.011), although sample sizes were smaller in the latter class and results were not strongly significant. No significant pattern was detected for other classes (Fig. S12b).

**Fig. 5:**
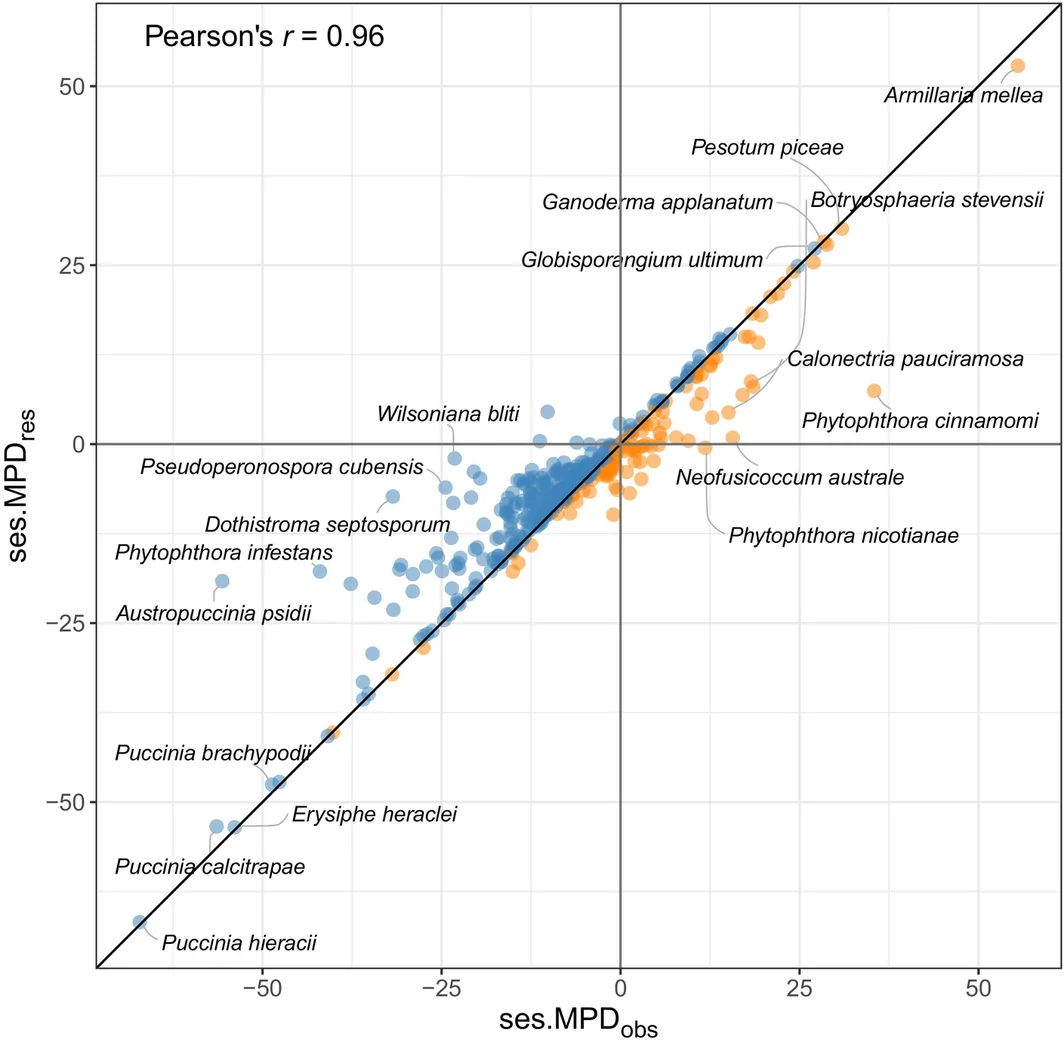
Change in phylogenetic host range breadth (ses.MPD; standardised effect size of Mean Pairwise Distance) for alien pathogens when considering all host associations (ses.MPD_obs_) vs excluding novel/co‐xenic associations (ses.MPD_restr_). Orange symbols below the diagonal indicate alien pathogens that extended their host range breadth through novel/co‐xenic associations (*n* = 114), while blue symbols above the diagonal indicate those taxa that acquired novel associations within the known phylogenetic host range breadth (*n* = 324). Host range breadth increases from left to right and from bottom to top. Negative ses.MPD values indicate smaller phylogenetic pairwise distances than expected (clustering), and positive values indicate greater phylogenetic pairwise distances than expected (dispersion). Taxa that are within the most extreme 1% of host range breadth extension/contraction or overall host range breadth are labelled. The number of taxa shown (*n* = 438 pathogen taxa) is smaller than the total number of taxa with novel associations because for some taxa ses.MPD_restr_ could not be calculated, as removal of novel host plants left fewer than two host species.

While 114 alien pathogens (26%) did have a wider host range breadth when including novel and co‐xenic associations (ses.MPD_obs_), the majority (324 taxa) acquired novel/co‐xenic associations within their known phylogenetic range. Overall, both measures (ses.MPD_obs_, i.e. including novel/co‐xenic associations and ses.MPD_restr_ excl. novel/co‐xenic associations) were highly correlated (Pearson's *r* = 0.96) (Fig. 5).

SR_obs_ and ses.MPD_obs_ showed only a weak correlation (Pearson's *r* = 0.15; Fig. S4), indicating limited association between taxonomic richness and phylogenetic host breadth. Consequently, taxa with the highest SR_obs_ and ses.MPD_obs_ have a moderate overlap – seven generalist taxa are found in the top 15 for both SR_obs_ and ses.MPD_obs_ (*Armillaria mellea*, *Ganoderma applanatum*, *Macrophomina phaseolina*, *P. cactorum*, *P. cinnamomi*, *P. cryptogea*, *P. hedraiandra*) (Table S7). Species with the strongest phylogenetic clustering (i.e. most negative ses.MPD_obs_) are dominated by Pucciniomycetes, particularly rust fungi (Pucciniales), and by Leotiomycetes, primarily powdery mildews (Erysiphaceae) (Fig. 5; Table S8).

### Correlates of novel and co‐xenic associations

**Fig. 6:**
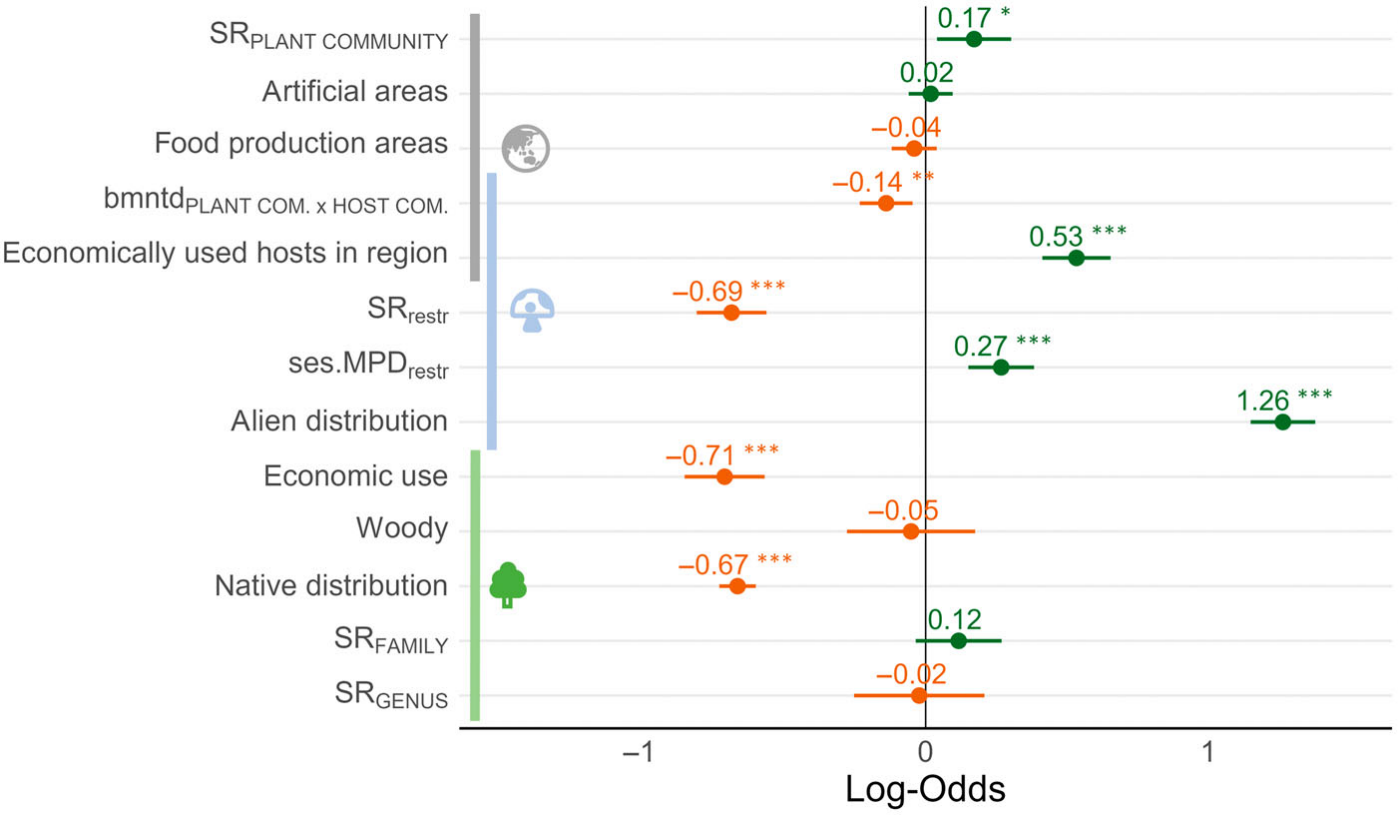
Fixed‐effect estimates (log‐odds) from the binomial generalised linear mixed model. Orange values indicate a negative, and dark green values indicate a positive effect on the probability that a region–pathogen–host combination is identified as novel/co‐xenic (*n* = 24 622). Error bars represent confidence intervals, and asterisks indicate the significance of the predictor (*, *P* < 0.05; **, *P* < 0.01; ***, *P* < 0.001). Crossed random effects are region (*n* = 201), pathogen genus (*n* = 375), and plant genus (*n* = 1630). Vertical bars and icons on the left indicate the predictor group (i.e. region‐, region–pathogen‐, pathogen‐, or plant‐related).

The likelihood of novelty decreased with beta mean nearest taxon distance between the regional plant and host community (bmntd_PLANT COM. × HOST COM._, −0.14 ± 0.05, *P* = 0.0034), and increased with the regions' overall plant richness (SR_PLANT COMMUNITY_, 0.17 ± 0.07, *P* = 0.0104), while the landcover predictors were not significant (Fig. 6; Table S9). At the pathogen level, records were more likely to be classified as novel/co‐xenic for taxa that were geographically widespread invaders (1.26 ± 0.06, *P* < 0.0001), showed a broader phylogenetic host range breadth (ses.MPD_restr_, 0.27 ± 0.06, *P* < 0.0001), and were present on other economically used plant hosts in the region (0.53 ± 0.06, *P* < 0.0001). Host SR (SR_restr_) showed a negative relationship (−0.69 ± 0.06, *P* < 0.0001) with novelty of association. At the host level, the likelihood of novelty decreased if the plant host was economically used (−0.71 ± 0.07, *P* < 0.0001) and with increasing native distribution (−0.67 ± 0.03, *P* < 0.0001). Plant genus‐ and family‐level SR as well as woodiness were not significant predictors (Figs S13–S15).

Sensitivity analyses confirmed that estimates were largely robust to alternative weighting schemes, random‐effect structures, and the exclusion of highly influential pathogen taxa (Figs S7, S8). Random effects indicated substantial variation among grouping levels, particularly for plant genera (SD = 2.58) and pathogen genera (SD = 1.96), whereas regional effects were comparatively smaller (SD = 0.74; Figs S16–S18). The full model including random factors explained 81.3% of the variation in the data (conditional *R* ^2^_GLMM_), with fixed effects alone accounting for 18.4% (marginal *R* ^2^_GLMM_).

## Discussion

Associations between plants and fungal and fungus‐like pathogens may play a key role in the spread and invasion success of both, with impacts ranging from emerging infectious diseases in native plants to reducing plant invasiveness (Anderson *et al*., 2004; Dickie *et al*., 2017). In this study, over a third of investigated pathogens formed at least one novel/co‐xenic association in their alien range, while the five most widespread generalist taxa accounted for a disproportionate share of more than one‐quarter of such identified pathogen–host associations. However, a large fraction of associations remained unclassified, highlighting that estimates of novelty are constrained by incomplete biogeographic and host information.

At the pathogen level, a broader restricted host range breadth (ses.MPD_restr_) was associated with novelty across classes, but significant within‐class differences between taxa forming novel/co‐xenic associations and those that did not were limited to two classes. However, a positive effect of ses.MPD_restr_ was also found at the record level. This suggests that a wider phylogenetic host range may facilitate emergence of novel/co‐xenic associations. However, a narrower phylogenetic host range does not necessarily constrain, as pathogens can still encounter suitable novel hosts during the invasion process, depending on the level of radiation within host plant clades and the phylogenetic structure of the recipient plant community. Consistent with phylogenetic conservatism in pathogen–host associations (Gougherty & Davies, 2021), most alien pathogens acquired novel/co‐xenic associations within their phylogenetic host range; however, around a quarter extended it.

In our model, novelty at the record level (i.e. region–pathogen–host association records) was strongly explained by a wider alien distribution of the pathogen and a restricted native range of the plant, suggesting that geographic barriers are major constraints on the formation of novel and co‐xenic associations. On the other hand, the negative relationship between novelty and the phylogenetic distance between the host community and regional plant community indicates an additional role of phylogenetic filtering. In line with this, a broader phylogenetic host range increased the likelihood of forming novel associations, highlighting the need to jointly consider geographic opportunity and host phylogenetic relatedness, when predicting and mitigating the risks of plant pathogen invasions.

### Novelty in plant pathogen invasions: a rare phenomenon?

Our findings indicate that novelty is not rare at the pathogen level, where over a third (36%) of the investigated pathogens showed at least one novel/co‐xenic association in their alien range. This suggests that pathogen‐mediated apparent competition may be a common source of indirect impacts of alien plants to native plants (Waller *et al*., 2024). These results are broadly comparable to findings in New Zealand (Bufford *et al*., 2016), where 27% of pathogens were recorded on at least one biogeographically novel host (considering novel associations of native and alien pathogens). However, that study focussed on a single, highly invaded and geographically isolated region and did not consider co‐xenic associations. These may further facilitate pathogen spread, particularly because alien plants are often widely introduced for economic use (van Kleunen *et al*., 2020). Indeed, widely cultivated species such as fruit trees harboured the highest numbers of pathogens, although this may partly reflect monitoring and reporting effort. Some associations, such as *P. cinnamomi* on *Banksia spp*., a genus native to Australia but alien in South Africa, were classified as both novel and co‐xenic, highlighting the dynamic nature of pathogen–plant associations during invasion processes. Reconstructing the sequence of these association types, which could arise independently or sequentially via alien‐to‐native host population shifts or vice versa, would provide insights into underlying spread dynamics and potential bridgehead effects.

Although live plant trade is a major introduction pathway for alien plant pathogens (Paap *et al*., 2022), we only identified very few unambiguous co‐introduced associations. While plant–pathogen invasions often begin with co‐introduction, particularly for generalist pathogens, newly acquired associations can become more prominent over time and further drive spread. By contrast, highly host‐specific pathogens rely on their co‐introduced host for spread, such as species of the smut genus *Entyloma*, which typically infect a single host and are known to be co‐introduced and spread with their ornamental host plants (Piątek *et al*., 2024). Pathogens may also be introduced independently via soil, timber, and other contaminated products (Hulme *et al*., 2008). However, the low number of identified co‐introductions likely rather reflects our conservative classification approach and limited information on biogeographic status and native geographic range, particularly for fungal and fungus‐like pathogens. Additionally, recording bias may further obscure co‐introductions, as novel/co‐xenic hosts often exhibit more obvious symptoms (Santini *et al*., 2018).

### Novel but on familiar grounds

The majority of alien pathogens exhibited phylogenetically clustered host ranges, consistent with studies showing that phylogenetic distance between plants can be a proxy for infection susceptibility by the same pathogen (Gilbert & Parker, 2016; Lynch *et al*., 2021). Novel/co‐xenic associations were predominantly recorded within the known phylogenetic host range of pathogens; however, around a quarter of pathogen taxa expanded their phylogenetic host range. Taxa showing such expansion include widespread generalists, such as members of *Phytophthora* (e.g. *P. cinnamomi* and *P. cambivora*), *Calonectria pauciramosa*, and root rot pathogens such as *Armillaria mellea*. By contrast, some successful invaders remain strongly phylogenetically constrained, such as the Myrtle rust (*A. psidii*), a member of the Pucciniales, which are biotrophic and typically considered specialists. Despite having one of the highest numbers of recorded novel/co‐xenic associations and infecting a wide range of host species, it remains restricted to members of the Myrtaceae across its introduced range (Soewarto *et al*., 2018).

Importantly, unidentified novel/co‐xenic associations may inflate our proxy for the initial phylogenetic host range breadth (ses.MPD_restr_). As a consequence, the number of pathogens inferred to acquire novel/co‐xenic associations within their phylogenetic host range may be overestimated. Therefore, our results present a conservative estimate of increases in phylogenetic host range breadth. Indeed, we found that around three times more alien pathogens acquired novel associations within their host range compared to those that extended their host range (324 vs 114 taxa). Disentangling the effect of phylogenetic conservatism vs methodological constraints remains a challenge, and clarification of true initial host ranges in alien pathogens' native ranges will be essential for refining estimates of increases in phylogenetic host range breadth.

Finally, while closely related plants are more likely to be colonised, occasional host shifts to more distantly related species may still occur through stochastic host encounters and increasing exposure over time (Roy, 2001). Here, improved data on time since introduction would help to better resolve the relative roles of phylogenetic constraints and temporal dynamics (Mitchell *et al*., 2010).

### Correlates on different organisational levels

Phylogenetic host range breadth showed a significant positive effect on the probability of a record being novel/co‐xenic, but this effect was modest compared to that of the pathogen's alien geographic range which may also partly reflect the time since initial spread. Thus, while phylogenetic host range breadth increases the likelihood of colonising novel hosts, geographic spread and resulting contact opportunities appear to be dominant drivers of novelty (but see Bufford *et al*., 2016, who investigated within‐region proxies for contact opportunity). Conversely, host generalism can promote spread and invasion success, yet specialists can also spread widely if their hosts are broadly distributed, as seen in staple crop pathogens (Scott *et al*., 2019). While observed host SR showed a weak association with the number of alien regions (*r* = 0.17), SR_restr_ showed no meaningful linear association (*r* = −0.04), suggesting that initial host range is not a major driver of alien range size. In our model, SR_restr_ showed a negative effect, which may partly reflect data limitations; the native range of many widespread generalist pathogens are unknown, and thus, they will be more likely to have more undefined associations and fewer classified novel/co‐xenic associations.

Regions with higher plant SR, and thus a larger potential host pool, were more likely to harbour novel/co‐xenic associations, although this effect was relatively weak compared with other significant predictors. By contrast, bmntd_PLANT COM. × HOST COM._ decreased the likelihood of novel/co‐xenic associations, in line with other findings that phylogenetic relatedness among plant species predicts the extent of shared pathogens (Gilbert *et al*., 2015). Potential host species in distant or isolated geographic regions will lack co‐evolutionary history with the pathogen or its relatives and, thus, have a lower degree of resistance (Thines, 2019). However, due to data limitations, we were not able to consider the geographic distance between native and introduced ranges, which may further structure these patterns.

Bufford *et al*. (2016) found that the number of congeners, but not confamiliars, significantly explained the number of novel associations for alien plant pathogens. In our model, after accounting for plant genus as a random effect, neither plant genus richness nor plant family richness showed a significant relationship with novelty. Random effects indicated considerable variation among plant and pathogen grouping levels, suggesting that baseline probabilities of novelty differ markedly among taxa. In addition, we did not detect a significant effect of host woodiness, despite expectations that woody plants may accumulate more pathogen diversity due to longevity and structural complexity. Both categories were well‐represented in the data underlying our model, with 57% of records pertaining to woody and 42% of records to nonwoody plants, suggesting that this lack of effect is not driven by sampling imbalance.

Raw wood and timber are known introduction pathways for pathogens of woody plants, possibly aided by vector insects that are associated with wood trade (Palm & Rossman, 2003; Wingfield *et al*., 2017). In addition, novel associations between native insect vectors and alien pathogens can further enhance transmission efficiency, often leading to the replacement of native fungi through niche occupation (Santini & Battisti, 2019). In general, unintentional transport as a contaminant of plants or plant products is thought to be a major pathway for pathogen introduction (Hulme *et al*., 2008; Sikes *et al*., 2018). Indeed, the presence of economically used hosts of a pathogen in the region increased the likelihood of novel/co‐xenic associations. On the other hand, noneconomically used, less widespread plants were more prone to novel/co‐xenic associations, highlighting risks to endemic plant species due to a lack of co‐evolutionary history. One example is New Zealand's iconic kauri (*Agathis australis*), which was among those species with the most‐recorded novel associations. However, emblematic endemic species may also experience intensified monitoring and might be more carefully studied than others.

While the fixed effects explained part of the variation in novelty (marginal *R* ^2^_GLMM_ = 0.18; conditional *R* ^2^_GLMM_ = 0.81), additional factors not included in the present analysis, including geographic distance from native ranges, climatic conditions, and time since introduction, may also contribute to variation in the occurrence of novel and co‐xenic associations.

### Pitfalls and potential limitations

Large‐scale syntheses across regions and taxa are useful for deriving new insights into general patterns (Dickey *et al*., 2021), but inevitably involve limitations. Specifically, the alien pathogen–host combinations examined here do not consider ecologically relevant factors such as disease severity or the abundance and co‐occurrence of original and potential hosts. Accurate assessment of association types and host ranges depends on precise species identification and comprehensive biogeographic knowledge (Desprez‐Loustau *et al*., 2007; Gladieux *et al*., 2015). Fungi are highly species‐rich, with much of their biodiversity still undescribed (Hawksworth & Lücking, 2017), and unsettled species concepts across sources can lead to inconsistent conclusions (Blanz & Zwetko, 2018). Host range indices may therefore be overestimated when based on outdated knowledge or inappropriate species concepts (Kruse *et al*., 2018), while incomplete sampling may lead to underestimation of natural host range.

Biodiversity data often show strong sampling bias (Hughes *et al*., 2021), particularly for inconspicuous organisms such as fungi, where detection probability varies strongly across space, time and host condition (Desprez‐Loustau *et al*., 2007; Bebber *et al*., 2019), and is thought to be higher in regions of introduction and on novel strongly symptomatic hosts. Similarly, pathogens on economically important or culturally valuable plants are more easily detected. Co‐introductions are likely harder to detect due to sparse native‐range data, less obvious impacts and endophytic behaviour in well‐adapted host plants, especially when both species originate from historically under‐sampled regions (Hughes *et al*., 2021). Association records in our dataset were unevenly distributed across space, with clear peaks in the United States and New Zealand (Fig. S3c,d).

We attempted to counteract those multidimensional sampling biases, at regional, pathogen, and plant level, using a weighting approach in our model. However, the requirement for calculation of ses.MPD_restr_ that at least two hosts must be present after excluding novel/co‐xenic associations, inevitably removed taxa with very high host specificity or poor sampling effort, potentially influencing estimates. In addition, pathogens frequently recorded on novel hosts, but with poorly known native host ranges are prone to misclassification of biogeographic status, while incomplete sampling of rare plants can also lead to underestimation of the pathogen host range breadth (Bufford *et al*., 2020). Precise delineation of association types requires reliable knowledge of the geographical ranges of both association partners, but specifically for fungi, this information is often missing (Desprez‐Loustau *et al*., 2007). Consequently, co‐xenic associations may be overestimated and co‐introductions underestimated when pathogen native ranges are incomplete or uncertain.

Finally, standardised, machine‐readable biodiversity data following frameworks such as Darwin Core (DwC), which enable consistent and thus interoperable recording (Wieczorek *et al*., 2012; Groom *et al*., 2019), are essential for large‐scale analyses of ecological interactions. However, many pathogen–host association records remain unstructured or inconsistently annotated, often lacking explicit interaction and biogeographic metadata, limiting their usability for global syntheses.

These points are reflected in the substantial proportion of pathogen–host records with undefined association type (46%), and a smaller subset in which novel and co‐xenic classifications were indistinguishable (6%) (Table S4). This uncertainty limits the resolution at which differences between association types can currently be evaluated at a global scale and may mask finer ecological or biogeographic patterns. Tackling these limitations will therefore be essential to refine future assessments of association novelty and underlying processes. Improved adherence to FAIR principles, broader adoption of standardised vocabularies, and stronger integration of biogeographic status and species interactions would substantially improve future studies. Despite these constraints, consistent patterns across organisational levels indicate the robustness of our main findings.

### Synthesis and implications

This study provides a global assessment of biogeographic novelty of alien pathogen–plant associations and their relationship to phylogenetic host range breadth. Drawing on the current state of knowledge, we find that across the spectrum of host specificity, novelty is a frequent phenomenon in alien pathogens. The majority of pathogen taxa acquired novel/co‐xenic associations within their known phylogenetic host range, highlighting the potential to assess the risk those pathogens pose to recipient plant communities. However, there was also a notable number of alien pathogens, including prominent taxa such as *P. cinnamomi*, which have expanded their phylogenetic host range to distantly related plant species. Thus, while the majority of novel associations are clustered, potential phylogenetic expansion of host range breadth remains a challenging task to quantify.

Across organisational levels, range‐related factors were the strongest predictors of recorded novelty. In particular, wider alien range of pathogens combined with narrower native distribution of host plant species increases the probability of novelty, underscoring the importance of contact opportunity as a prerequisite for novel associations. Phylogenetic similarity between host communities and recipient plant communities, and a broader phylogenetic host range further promote novelty. Knowledge of phylogenetic host range and past host range extensions can help to rapidly screen the spillover risk of alien pathogens to new plant species in recipient regions (Gilbert *et al*., 2015), providing a basis for more detailed analyses. However, pathogen virulence is association‐specific and context‐dependent, and some taxa may shift between lifestyles, from prominent pathogens to harmless endophytes and vice versa. Accordingly, our study does not allow for drawing inferences on the impacts of alien pathogens on plants, which depends on the pathogen itself, the susceptibility of the hosts and the conduciveness of the environment (disease triangle) (Scholthof, 2007; Méthot & Alizon, 2014).

Further information on ecological impacts, coupled with association information, can give valuable insights into the actual and potential consequences associated with novel/co‐xenic associations of alien pathogens. Future work should also focus on understanding the role of associated functional traits. For example, necrotrophic pathogens (i.e. those that kill tissue before extracting nutrients) are thought to more frequently experience host jumps (Delaye *et al*., 2013). Root‐attacking members of the genus *Phytophthora* tend to be more widespread and have a broader host range than their foliar congeners (Barwell *et al*., 2020; Marcot *et al*., 2023). Linking such traits to global patterns could help identify invasion syndromes (*sensu* Novoa *et al*., 2020) in alien pathogens and thus inform risk assessment and species management.

Finally, a considerable proportion of associations in the pathogen's introduced range could not be unambiguously classified, highlighting the importance of addressing such knowledge gaps in fungal biogeography in future research. Phylogeographic studies may provide an improved baseline for studying ecological novelty during pathogen invasions. An improved knowledge of the phenomenon of ecological novelty in alien pathogen–plant associations will be important as we seek to better understand the associated risks of fungal and fungus‐like pathogen invasions.

## Competing interests

None declared.

## Author contributions

A Schertler, BL, SD, DM and FE planned and designed the research. A Schertler compiled the dataset with contributions to pathogen data from JLB, A Santini, LG, PT, and to plant data from WD, MK, HK, JP, PP, PW, MW and FE. Feedback for pathogen data validation was provided by IK‐G, HV, A Santini, LG, PT, MJW and MT. A Schertler analysed the data with contributions from BL, SD, DM and AG‐R. A Schertler wrote the manuscript, with significant contributions from all co‐authors (AG‐R, A Santini, BL, CC, DM, FE, HK, HV, IK‐G, JLB, JP, LG, LR, MK, MJW, MT, MW, PP, PT, PW, SD, WD).

## Disclaimer

The New Phytologist Foundation remains neutral with regard to jurisdictional claims in maps and in any institutional affiliations.

## Supporting information


**Fig. S1** Detailed study workflow.
**Fig. S2** Spatial units used in this study.
**Fig. S3** Data coverage across regions.
**Fig. S4** Correlations among host diversity metrics.
**Fig. S5** Schematic of the calculation of ses.MPD.
**Fig. S6** Correlations among GLMM predictors.
**Fig. S7** Sensitivity of fixed‐effect estimates to alternative model specifications.
**Fig. S8** Sensitivity of fixed‐effect estimates to exclusion of dominant pathogen taxa.
**Fig. S9** Novelty by higher taxonomic group.
**Fig. S10** Plant families most represented as hosts of alien pathogens.
**Fig. S11** Phylogenetic indices of restricted host range breadth by class: between‐class differences.
**Fig. S12** Phylogenetic indices of restricted host range breadth by class: within‐class differences in novelty.
**Fig. S13** Marginal effects by region.
**Fig. S14** Marginal effects by pathogen genus.
**Fig. S15** Marginal effects by plant genus.
**Fig. S16** Random effects by region.
**Fig. S17** Random effects by pathogen genus.
**Fig. S18** Random effects by plant genus.
**Methods S1** Additional information on data sources and data preparation.
**Table S1** Pathogen taxa removed for taxonomy uncertainty.
**Table S2** Sources used in the final datasets.
**Table S3** Biogeographic status of pathogens and their hosts across regions.
**Table S4** Association types in pathogens' alien ranges.
**Table S5** Top 25 plant species by pathogen richness across association types.
**Table S6** Clustered vs dispersed observed phylogenetic host ranges across fungal classes.
**Table S7** The 15 taxa with broadest observed host ranges.
**Table S8** The 15 taxa with phylogenetically most constrained host ranges (lowest ses.MPD_obs_).
**Table S9** Summary of the binomial generalised linear mixed model.
Please note: Wiley is not responsible for the content or functionality of any Supporting Information supplied by the authors. Any queries (other than missing material) should be directed to the *New Phytologist* Central Office.

## Data Availability

The data and code supporting the findings of this study are available in a Zenodo repository at doi: 10.5281/zenodo.17271581.
