# Supplementary material for: Are novel and co‐xenic associations common in alien fungal and fungus‐like plant pathogens?
Source: New Phytol. 2026 Aug 24;252(2):843–60. doi: 10.1111/nph.71455 (PMC13583412; doi:10.1111/nph.71455)
Supplement: Supplementary file 1 [file NPH-252-843-s001.docx]

## *New Phytologist* Supporting Information

Article title: Are novel and co-xenic associations common in alien fungal and fungus-like plant pathogens?

Authors: Anna Schertler*, Bernd Lenzner, Stefan Dullinger, Dietmar Moser, Adrián García-Rodríguez, Irmgard Krisai-Greilhuber, Hermann Voglmayr, Jennifer L. Bufford, Alberto Santini, Luisa Ghelardini, César Capinha, Luís Reino, Michael J. Wingfield, Marco Thines, Pedro Talhinhas, Wayne Dawson, Mark van Kleunen, Holger Kreft, Jan Pergl, Petr Pyšek, Patrick Weigelt, Marten Winter*,* Franz Essl

*Corresponding author
Email: anna.schertler@univie.ac.at

Article acceptance date: 1 July 2026

**Contents**

**Supporting Figures and Tables**	**3**

**Figures**	**3**

Fig. S1 Detailed study workflow	3

Fig. S2 Spatial units used in this study	4

Fig. S3 Data coverage across regions	4

Fig. S4 Correlations among host diversity metrics	5

Fig. S5 Schematic of the calculation of ses.MPD.	6

Fig. S6 Correlations among GLMM predictors.	7

Fig. S7 Sensitivity of fixed-effect estimates to alternative model specifications.	8

Fig. S8 Sensitivity of fixed-effect estimates to exclusion of dominant pathogen taxa	9

Fig. S9 Novelty by higher taxonomic group	10

Fig. S10 Plant families most represented as hosts of alien pathogens	11

Fig. S11 Phylogenetic indices of restricted host range breadth by class: between-class differences...	12

Fig. S12 Phylogenetic indices of restricted host range breadth by class: within-class differences in novelty	13

Fig. S13 Marginal effects by region	14

Fig. S14 Marginal effects by pathogen genus	15

Fig. S15 Marginal effects by plant genus	16

Fig. S16 Random effects by region.	17

Fig. S17 Random effects by pathogen genus.	18

Fig. S18 Random effects by plant genus	19

**Tables**	**20**

Table S1 Pathogen taxa removed for taxonomy uncertainty..	20

Table S2 Sources used in the final datasets.	21

Table S3 Biogeographic status of pathogens and their hosts across regions.	23

Table S4 Association types in pathogens‘ alien ranges	23

Table S5 Top 25 plant species by pathogen richness across association types.	24

Table S6 Clustered versus dispersed observed phylogenetic host ranges across fungal classes	25

Table S7 The 15 taxa with broadest observed host ranges	26

Table S8 The 15 taxa with phylogenetically most constrained observed host ranges (lowest ses.MPD_obs_).	26

Table S9 Summary of the binomial generalised linear mixed model.	27

**Methods S1 - Additional information on data sources and data preparation	28
Global Database of Alien Pathogenic Fungi	33**

Distribution ranges	33

Associations globally and on regional level	34

**Plant taxonomy and phylogeny	34
Plant distribution	35**

GIFT	35

GloNAF	36

WCVP	37

Combining the sources	38

**Modelling	39**

Weights	39

Random effects structure	40

Sensitivity analyses	41

**References	42**

# **Supporting Figures and Tables**

***Fig. S1*** ***Detailed study workflow.*** *Original data sources (pathogen data in orange and plant data in green) were standardised and aggregated using the World Catalogue of Vascular Plants (WCVP, Govaerts et al., 2025) and a shapefile containing the study regions, mapped in Fig. S2. After taxonomic and spatial aggregation, the biogeographic status of plants derived from the different sources was harmonised. Derived datasets used for further analyses are shown in dark grey. Abbreviations: BMNTD = Beta Mean Nearest Taxon Distance; GIFT = Global Inventory of Floras and Traits; GloNAF = Global Naturalized Alien Flora; LC = Land Cover; ses.MPD = standardised effect size of Mean Pairwise Distance; SR = Species richness; WCVP = World Checklist of Vascular Plants.*

***Fig. S2*** ***Spatial units used in this study.*** *Spatial units are contiguous countries or islands/archipelagos based on the Database of Global Administrative Areas (GADM V.3.6, https://gadm.org/) and the World Geographical Scheme for Recording Plant Distributions (TDWG; Brummitt, 2001). Countries comprising geographically separated units, such as oceanic islands, were split accordingly (e.g. Spain and the Canary Islands, United States and Hawai’i).*

***Fig. S3*** ***Data coverage across regions.*** *a) plant species richness, b) alien pathogens total distribution records (including unknown, native and alien/cryptogenic records), c) all associations records, d) alien range association records.*

***Fig. S4*** ***Correlations among host diversity metrics.*** *Correlation plot* *(Pearson’s r) for observed SR (host species richness), PD (host phylogenetic diversity) and ses.MPD (standardized effect size of host phylogenetic divergence, based on mean pairwise phylogenetic distance).*

***Fig. S5*** ***Schematic of the calculation of ses.MPD.*** *For each pathogen the observed mean pairwise phylogenetic distance (MPD) among recorded hosts was computed, then compared to a null distribution generated by randomising tips within the potential source pool (= all plant species occurring within the pathogen’s regional range) and pruning the randomised tree to the same host count. This was repeated 999 times to obtain. MPD_null_ and SD_null_. The standardised effect size was calculated as ses.MPD = (MPD - MPD_null_)/SD_null_. This was done twice, once for the full host community and once for the host community subset that excluded the novel and co-xenic associations.*

***Fig. S6*** ***Correlations among GLMM predictors.*** *Correlation* *plot (Pearson’s r) for the numeric and binary predictors used in the Generalised Linear Mixed Model. No predictor pair correlations were exceeding |r| > 0.6.*

***Fig. S7*** ***Sensitivity of fixed-effect estimates to alternative model specifications.*** *Coefficient estimates (log-odds scale) and 95% confidence intervals (CI) from the main Generalised Linear Mixed Model are compared with alternative model formulations, including uncapped weights, different weight-capping thresholds (99.9% and 99%), alternative random-effect structures (higher taxonomic levels, i.e. plant order and pathogen class), and a model without weights. Estimates are shown for the predictors included in the model.*

***Fig. S8*** ***Sensitivity of fixed-effect estimates to exclusion of dominant pathogen taxa.*** *Coefficient estimates (log-odds scale) and 95% confidence intervals from the main Generalised Linear Mixed Model are compared with models excluding the top 1% (2 taxa, accounting for ~10% of observations) and 2.5% (4 taxa, accounting for ~14% of observations) of the most frequently recorded pathogen taxa.*

***Fig. S9*** ***Novelty by higher taxonomic group.*** *Balloon plot showing counts of pathogen taxa with (TRUE) and without (FALSE) novelty in their host associations, grouped by class and phylum. Circle size and colour indicate the number of pathogen taxa. Here, ‘novelty’ indicates taxa with at least one novel/co-xenic host association.*

***Fig. S10*** ***Plant families most represented as hosts of alien pathogens.*** *Shown are the top 25 plant families by number of host species for all a) all associations, b) novel- and co-xenic novel associations, and c) novel associations only. The colour of bars indicates the proportion of economically used (orange) vs. not used plants (purple), considering all types of uses.*

***Fig. S11*** ***Phylogenetic indices of restricted host range breadth by class: between***-***class differences.*** *Boxplots of* ***a)*** *restricted host species richness SR_restr_ (N = 1248) and* ***b)*** *restricted host phylogenetic divergence ses.MPD_restr_ (standardised effect size of Mean Pairwise Distance; N = 1191) across 17 classes in five phyla. Taxa are grouped into those with identified novel or co-xenic associations (red) and those with none (blue). Incertae sedis taxa are pooled and shown for completeness but were excluded from statistical tests due to ambiguous taxonomic placement. Class names are coloured after phylum: green = Ascomycota, yellow = Basidiomycota, red = Blastocladiomycota, dark purple = Chytridiomycota, blue = Oomycota. The boxplots are accompanied by statistical test results on group differences between fungal classes (Wilcoxon post hoc pairwise comparisons). Grey bars and the associated asterisks indicate significant differences (*, p < 0.05; **, p < 0.01; ***, p > 0.001). Overall difference is indicated by results of a Kruskal-Wallis rank sum test. Classes represented by a single taxon are shown in the figure but were excluded from statistical analyses.*

***Fig. S12*** ***Phylogenetic indices of restricted host range breadth by class: within-class differences in novelty.*** *Boxplots of* ***a)*** *Restricted host species richness SR_restr_ (N = 1248) and* ***b)*** *restricted host phylogenetic divergence ses.MPD_restr_ (standardised effect size of Mean Pairwise Distance; N = 1191) for 17 classes in five phyla, with taxa being grouped into those with identified novel or co-xenic associations (red) and those with none (blue). Incertae sedis taxa are pooled and shown for completeness but were excluded from statistical tests due to their ambiguous taxonomic placement. Class names are coloured after phylum: green = Ascomycota, yellow = Basidiomycota, red = Blastocladiomycota, dark purple = Chytridiomycota, blue = Oomycota. The boxplots are accompanied with statistical test results on differences in novelty within fungal classes (Wilcoxon post hoc pairwise comparison). Grey bars and the associated asterisks indicate significant differences (*, p < 0.05; **, p < 0.01; ***, p > 0.001). Overall difference is indicated by results of a Kruskal-Wallis rank sum test.*

***Fig. S13*** ***Marginal effects by region.*** *Regions (n = 201) are coloured by continent, with all other predictors held constant. Black lines/dots show population-level predictions, i.e. across regions, while coloured lines/dots show region-specific predictions.*

***Fig. S14 Marginal effects by pathogen genus.*** *Genera (n = 373) are coloured by phylum, with all other predictors held constant. Black lines/dots show population-level predictions, i.e. across genera, while coloured lines/dots show the genus-specific predictions.*

***Fig. S15*** ***Marginal effects by plant genus.*** *Plant genera (n = 1630) are shown with all other predictors held constant. Black lines/dots show population-level predictions, i.e. across genera, while coloured lines/dots represent the genus-specific predictions.*

***Fig. S16*** ***Random effects by region.*** *Blue lines indicate positive and red lines negative effects.*

***Fig. S17*** ***Random effects by pathogen genus.*** *Blue lines indicate positive and red lines negative effects.*

**Fig. S18** ***Random effects by plant genus****. Blue lines indicate positive and red lines negative effects.*

**Tables**

***Table S1 Pathogen taxa removed for taxonomy uncertainty****. Taxa were excluded when species-level identification was considered unreliable due to known taxonomic issues, including cryptic species, species complexes, sensu lato usage, or documented systematic misidentifications.*

| **Taxon** | **Reference** |
| --- | --- |
| *Acremonium zonatum* | Hou et al. 2023. Stud. Mycol. 105:23–203 |
| *Albugo candida* | Choi et al. 2011. Fungal Biol. 115:598–607 |
| *Alternaria spp.* | Woudenberg et al. 2015. Stud. Mycol. 82:1–21 |
| *Basidiophora entospora* | Davis & Crouch. 2022. Fungal Ecol. 55:101126 |
| *Berkeleyomyces basicola* | Nel et al. 2018. Plant Pathol. 67:871–882 |
| *Blumeria graminis* | Liu et al. 2021. Mycoscience 62:143–165 |
| *Boeremia exigua* | Aveskamp et al. 2009. Mol. Plant Pathol. 10:403–414 |
| *Botrytis cinereal* | Garfinkel. 2021. Phytopathology 111:437–454 |
| *Calonectria pseudonaviculata* | LeBlanc et al. 2018. Appl. Microbiol. Biotechnol. 102: 4371–4380 |
| *Ceratocystis fimbriata* | de Beer et al. 2014. Stud. Mycol. 79:187–219 |
| *Ceratocystis paradoxa* | Mbenoun et al. 2014. Mycologia 106:757–784 |
| *Cercospora apii* | Groenewald et al. 2005. Phytopathology 95: 951–959 |
| *Cercospora beticola* | Vaghefi et al. 2018. Fungal Biol. 122:264–282 |
| *Ciborinia allii* | CAB International. 2021. Invasive Species Compendium ISC |
| *Claviceps purpurea* | Pažoutová et al. 2015. Fungal Biol. 119: 7–26 |
| *Coleosporium asterum* | McTaggart & Aime. 2018. Fungal Biol. 122: 800–809 |
| *Coleosporium tussilaginis* | Blanz & Zwetko. 2018. In: Biodiv. and Ecol. of Fungi, Lichens, and Mosses. p289–310 |
| *Cytospora ceratosperma* | H Voglmayr (pers. comm.) |
| *Entyloma compositarum* | H Voglmayr (pers. comm.) |
| *Entyloma ranunculi-repentis* | Kruse et al. 2018. Persoonia 41:175–201 |
| Fusarium species complexes:  *Fusarium chlamydosporum, F. equiseti, F. fujikuroi, F. graminearum, F. incarnatum, F. lateritium, F. oxysporum, F. sambucinum, F. tricinctum* | Crous et al. 2021. Stud. Mycol. 98:1–184.;  fusarium.org (2026) |
| *Fusarium roseum* | Summerell. 2019. Annu. Rev. Phytopathol. 57:323–339 |
| *Golovinomyces orontii* | U. Braun (pers. comm.) |
| *Heterobasidion annosum* | Chen et al. 2015. Fungal Diversity 71:185–200 |
| *Leveillula taurica* | Khodaparast et al. 2001. Mycol. Res. 8:909–918 |
| *Melampsora epitea* | Zhao et al. 2017. Mycologia 109:391–401 |
| *Melanconium atrum* | H Voglmayr (pers. comm.) |
| *Microbotryum violaceum* | Lutz et al. 2008. Mycol. Res. 112:1280–1296 |
| *Neocosmospora solani*  *(Syn. Fusarium solani species complex)* | Schroers et al. 2016. Mycologia 108: 806–819;  Sandoval-Denis et al. 2019. Persoonia 43: 90–185 |
| *Peronospora alta* | H. Voglmayr (pers. comm.) |
| *Phakopsora pachyrhizi* | Goellner et al. 2010. Mol. Plant Pathol. 11:169–177 |
| *Phytophthora citricola* | Jung & Burgess. 2009. Persoonia. 22:95–110 |
| *Puccinia coronata* | Liu & Hambleton. 2013a. Mycol. Prog. 12:63–89 |
| *Puccinia graminis* | Abbasi et al. 2005. Mycoscience 46:241–247 |
| *Puccinia recondita* | Blanz & Zwetko. 2018. In: Biodiv. and Ecol. of Fungi, Lichens, and Mosses. p289–310. |
| *Puccinia striiformis* | Chen. 2005. Can. J. Plant Pathol. 27:314–337 |
| *Pyrrhoderma noxium* | Bragard et al. 2024. EFSA J 22 |
| *Rhizoctonia solani* | Ajayi-Oyetunde & Bradley. 2018. Plant Pathol. 67:3–17. |
| *Sawadea bicornis* | U. Braun (pers. comm.) |
| *Sawadeae tulasnei* | U. Braun (pers. comm.) |
| *Urocystis agropyri, U. tritici* | Savchenko et al. 2017. Plant Pathol. 66: 1139–1148. |
| *Ustilago bullata* | H. Voglmayr (pers. comm); McKenzie & Vánky 2001. N. Z. J. Bot. 39:501–515. |
| *Ustilago striiformis* | Kruse et al. 2018. IMA Fungus 9:49–73. |
| *Verticillium albo-atrum* | EFSA PLH. 2014. EFSA J 12:3927. |

***Table S2 Sources used in the final datasets.*** *Distribution (D) and association (A) columns indicate whether the source was used those final datasets.* **) Please note RBG Kew cannot warrant the quality or accuracy of the data.*

| **Reference** | **DOI/url** | **D** | **A** |
| --- | --- | --- | --- |
| Afonin et al. 2008. Agroatlas. | https://www.agroatlas.ru | x | x |
| Arechavaleta et al. 2005. Lista preliminar de especies silvestres de Cabo Verde. Gobierno de Canarias. 155 pp. |  | x |  |
| Arechavaleta et al. 2010. Lista de especies silvestres de Canarias. Gobierno de Canarias. | https://www.biodiversidadcanarias.es/biota | x |  |
| Barwell et al. 2020. Phytophthora global impacts v1.0.0. Zenodo. | https://doi.org/10.5281/zenodo.4081474 | x | x |
| Batista et al. 2021. Forests 12(3):313. | https://doi.org/10.3390/f12030313 | x | x |
| Baudin et al. 2024. Mol. Plant Pathol. 25(4):e13449. | https://doi.org/10.1111/mpp.13449 | x | x |
| Broders et al. 2015. Forest Pathol. 45(1):1–8. | https://doi.org/10.1111/efp.12161 | x | x |
| CAB International 2020. Invasive Species Compendium. | https://www.cabi.org/isc/ | x |  |
| CBS-KNAW 2020. CBS Strain Database. | collected through correspondence | x | x |
| Carnegie & Pegg 2018. Annu. Rev. Phytopathol. 56:457–478. | https://doi.org/10.1146/annurev-phyto-080516-035256 | x | x |
| DeLong et al. 2019. PeerJ 7:e7986. | https://doi.org/10.7717/peerj.7986 | x | x |
| Desprez-Loustau et al. 2010. Biol. Invasions 12(1):157–172. | https://doi.org/10.1007/s10530-009-9439-y | x | x |
| EPPO 2020. EPPO Global Database. | https://gd.eppo.int | x | x |
| Farr & Rossman 2022. Fungal Databases, U.S. National Fungus Collections. | https://nt.ars-grin.gov/fungaldatabases/ | x | x |
| Farr et al. 2021. United States National Fungus Collections Fungus-Host Dataset. Ag Data Commons. | https://doi.org/10.15482/USDA.ADC/1524414 | x | x |
| Zeddam et al. 2020. GRIIS Algeria v1.9. | https://cloud.gbif.org/griis/resource?r=griis-algeria&v=1.9 | x |  |
| Togia et al. 2023. GRIIS American Samoa v1.7. | https://cloud.gbif.org/griis/resource?r=american-samoa_griis_gbif&v=1.7 | x |  |
| Salas Sopena et al. 2020. GRIIS Andorra v1.8. | https://cloud.gbif.org/griis/resource?r=griis-andorra&v=1.8 | x |  |
| Zalba et al. 2021. GRIIS Argentina v1.6. | https://cloud.gbif.org/griis/resource?r=griis-argentina&v=1.6 | x |  |
| Randall et al. 2022. GRIIS Australia v1.9. | https://cloud.gbif.org/griis/resource?r=griis-australia&v=1.9 | x |  |
| Pagad 2020. GRIIS Lord Howe Island (Australia) v1.0. | https://cloud.gbif.org/griis/resource?r=pa-griis-lord-howe&v=1.0 | x |  |
| Essl et al. 2020. GRIIS Austria v1.5. | https://cloud.gbif.org/griis/resource?r=griis-austria&v=1.5 | x |  |
| Pagad & Wong 2022. GRIIS Azerbaijan v1.1. | https://cloud.gbif.org/griis/resource?r=griis-azerbaijan&v=1.1 | x |  |
| Desmet et al. 2023. GRIIS Belgium v1.13. | https://doi.org/10.15468/xoidmd | x |  |
| Pagad 2022. GRIIS Bolivia v1.3. | https://cloud.gbif.org/griis/resource?r=griis-bolivia&v=1.3 | x |  |
| Maslo et al. 2020. GRIIS Bosnia and Herzegovina v1.3. | https://cloud.gbif.org/griis/resource?r=griis-bosnia-and-herzegovina&v=1.3 | x |  |
| Ziller et al. 2020. GRIIS Brazil v1.5. | https://cloud.gbif.org/griis/resource?r=brazil-griis-gbif&v=1.5 | x |  |
| Rodríguez Luengo et al. 2020. GRIIS El Hierro (Spain) v1.5. | https://cloud.gbif.org/griis/resource?r=griis-spain-canaris-el-hierro&v=1.5 | x |  |
| Rodríguez Luengo et al. 2020. GRIIS Gran Canaria (Spain) v1.6. | https://cloud.gbif.org/griis/resource?r=griis-canarias-gran-canaria&v=1.6 | x |  |
| Rodríguez Luengo et al. 2020. GRIIS Lanzarote (Spain) v1.5. | https://cloud.gbif.org/griis/resource?r=griis-canarias-lanzarote&v=1.5 | x |  |
| Rodríguez Luengo et al. 2020. GRIIS Tenerife (Spain) v1.4. | https://cloud.gbif.org/griis/resource?r=griis-tenerife-islas-canarias&v=1.4 | x |  |
| Rodríguez Luengo et al. 2020. GRIIS La Gomera (Spain) v1.6. | https://cloud.gbif.org/griis/resource?r=griis-canarias-la-gomera&v=1.6 | x |  |
| Rodríguez Luengo et al. 2020. GRIIS La Palma (Spain) v1.7. | https://cloud.gbif.org/griis/resource?r=griis-canarias-la-palma&v=1.7 | x |  |
| Pauchard et al. 2020. GRIIS Chile v2.7. | https://doi.org/10.15468/n4ofia | x |  |
| Zhao et al. 2020. GRIIS China v1.3. | https://cloud.gbif.org/griis/resource?r=griis-china&v=1.3 | x |  |
| Piedad Baptiste et al. 2022. GRIIS Colombia v1.7. | https://cloud.gbif.org/griis/resource?r=griis-colombia&v=1.7 | x |  |
| Pagad & Wong 2022. GRIIS Congo v1.1. | https://cloud.gbif.org/griis/resource?r=griis-cr&v=1.1 | x |  |
| McCormack et al. 2023. GRIIS Cook Islands v2.12. | https://doi.org/10.15468/tilinz | x |  |
| Gonzalez-Oliva et al. 2023. GRIIS Cuba v2.10. | https://doi.org/10.15468/jzyeh0 | x |  |
| Martinou et al. 2020. GRIIS Cyprus v1.9. | https://cloud.gbif.org/griis/resource?r=griis-cyprus&v=1.9 | x |  |
| Müller et al. 2020. GRIIS Denmark v1.6. | https://cloud.gbif.org/griis/resource?r=griis-denmark&v=1.6 | x |  |
| Pagad & Wong 2022. GRIIS Dominican Republic v1.1. | https://cloud.gbif.org/griis/resource?r=griis-dom-rep&v=1.1 | x |  |
| Vunk et al. 2020. GRIIS Estonia v1.2. | https://cloud.gbif.org/griis/resource?r=griis-estonia&v=1.2 | x |  |
| Pagad 2022. GRIIS Fiji v1.2. | https://cloud.gbif.org/griis/resource?r=griis-fiji&v=1.2 | x |  |
| Niemivuo-Lahti et al. 2020. GRIIS Finland v1.3. | https://cloud.gbif.org/griis/resource?r=griis-finland&v=1.3 | x |  |
| Thevenot et al. 2022. GRIIS France v1.6. | https://cloud.gbif.org/griis/resource?r=griis-france&v=1.6 | x |  |
| Causton et al. 2020. GRIIS Galápagos Islands v1.5. | https://cloud.gbif.org/griis/resource?r=griis_galapagos_islands&v=1.5 | x |  |
| Pagad 2020. GRIIS Galápagos Islands (protected areas) v1.0. | https://cloud.gbif.org/griis/resource?r=pa-griis-galapagos&v=1.0 | x |  |
| Kolbaia et al. 2020. GRIIS Georgia v1.5. | https://cloud.gbif.org/griis/resource?r=griis-georgia&v=1.5 | x |  |
| Gollasch et al. 2022. GRIIS Germany v1.6. | https://cloud.gbif.org/griis/resource?r=griis-germany&v=1.6 | x |  |
| Pagad 2022. GRIIS Guatemala v1.1. | https://cloud.gbif.org/griis/resource?r=griis-gautemala&v=1.1 | x |  |
| Sankaran et al. 2021. GRIIS India v1.5. | https://cloud.gbif.org/griis/resource?r=griis-india&v=1.5 | x |  |
| Setyawati et al. 2020. GRIIS Indonesia v1.1. | https://cloud.gbif.org/griis/resource?r=griis-indonesia&v=1.1 | x |  |
| Keshavarzi et al. 2020. GRIIS Iran v1.2. | https://cloud.gbif.org/griis/resource?r=griis-iran&v=1.2 | x |  |
| O'Flynn et al. 2020. GRIIS Ireland v2.7. | https://doi.org/10.15468/br8rgw | x |  |
| Dufour-Dror et al. 2020. GRIIS Israel v1.3. | https://cloud.gbif.org/griis/resource?r=griis-israel&v=1.3 | x |  |
| Donaldson et al. 2020. GRIIS Jamaica v1.2. | https://cloud.gbif.org/griis/resource?r=griis-jamaica&v=1.2 | x |  |
| Pagad 2020. GRIIS Kazakhstan v1.1. | https://cloud.gbif.org/griis/resource?r=griis-kazakhstan&v=1.1 | x |  |
| Witt et al. 2020. GRIIS Kenya v1.4. | https://cloud.gbif.org/griis/resource?r=griis-kenya&v=1.4 | x |  |
| Balalaikins & Pagad 2020. GRIIS Latvia v1.3. | https://cloud.gbif.org/griis/resource?r=griis-latvia&v=1.3 | x |  |
| Mueller et al. 2020. GRIIS Liechtenstein v1.2. | https://cloud.gbif.org/griis/resource?r=griis-liechtenstein&v=1.2 | x |  |
| Ries & Pagad 2020. GRIIS Luxembourg v1.2. | https://cloud.gbif.org/griis/resource?r=griis-luxembourg&v=1.2 | x |  |
| Tataya et al. 2020. GRIIS Mauritius v1.1. | https://cloud.gbif.org/griis/resource?r=griis-mauritius&v=1.1 | x |  |
| González Martínez et al. 2020. GRIIS Mexico v1.5. | https://cloud.gbif.org/griis/resource?r=griis-mexico&v=1.5 | x |  |
| Moverly et al. 2020. GRIIS Micronesia v1.4. | https://cloud.gbif.org/griis/resource?r=griis-federated-states-of-micronesia&v=1.4 | x |  |
| Nistreanu et al. 2020. GRIIS Moldova v1.4. | https://cloud.gbif.org/griis/resource?r=griis-moldova&v=1.4 | x |  |
| Trajanovski et al. 2020. GRIIS North Macedonia v1.3. | https://cloud.gbif.org/griis/resource?r=griis-macedonia&v=1.3 | x |  |
| Hilmo et al. 2021. GRIIS Norway v1.15. | https://cloud.gbif.org/griis/resource?r=griis-brunei-norway&v=1.15 | x |  |
| Orapa & Pagad 2020. GRIIS Papua New Guinea v1.2. | https://cloud.gbif.org/griis/resource?r=griis-papua-new-guinea&v=1.2 | x |  |
| Pagad 2022. GRIIS Peru v1.2. | https://cloud.gbif.org/griis/resource?r=griis-peru&v=1.2 | x |  |
| Solarz et al. 2020. GRIIS Poland v1.3. | https://cloud.gbif.org/griis/resource?r=griis-poland&v=1.3 | x |  |
| Cogălniceanu et al. 2020. GRIIS Romania v1.3. | https://cloud.gbif.org/griis/resource?r=griis-romania&v=1.3 | x |  |
| Petrosyan et al. 2020. GRIIS Russian Federation v2.7. | https://doi.org/10.15468/f6joyb | x |  |
| Rat et al. 2020. GRIIS Serbia v1.2. | https://cloud.gbif.org/griis/resource?r=griis-serbia&v=1.2 | x |  |
| Senterre et al. 2023. GRIIS Seychelles v2.14. | https://doi.org/10.15468/9e9pfi | x |  |
| Pagad 2022. GRIIS Slovakia v1.1. | https://cloud.gbif.org/griis/resource?r=griis-slovakia&v=1.1 | x |  |
| De Groot et al. 2020. GRIIS Slovenia v1.2. | https://cloud.gbif.org/griis/resource?r=griis-slovenia&v=1.2 | x |  |
| Foxcroft et al. 2020. GRIIS Golden Gate Highlands NP (South Africa) v1.1. | https://cloud.gbif.org/griis/resource?r=pa-griis-golden-gate&v=1.1 | x |  |
| Robinson et al. 2020. GRIIS South Africa v2.7. | https://doi.org/10.15468/l6smob | x |  |
| Foxcroft et al. 2020. GRIIS Kruger NP (South Africa) v1.1. | https://cloud.gbif.org/griis/resource?r=pa-griis-kruger&v=1.1 | x |  |
| Pagad et al. 2022. GRIIS Sweden v1.2. | https://cloud.gbif.org/griis/resource?r=griis-sweden&v=1.2 | x |  |
| Weber et al. 2020. GRIIS Switzerland v1.2. | https://cloud.gbif.org/griis/resource?r=griis-switzerland&v=1.2 | x |  |
| Pagad 2022. GRIIS Trinidad and Tobago v1.2. | https://cloud.gbif.org/griis/resource?r=griis-trinidad-tobago&v=1.2 | x |  |
| Bouktila et al. 2020. GRIIS Tunisia v2.7. | https://doi.org/10.15468/hbprcp | x |  |
| Roy et al. 2020. GRIIS Great Britain v1.7. | https://cloud.gbif.org/griis/resource?r=griis-united_kingdom&v=1.7 | x |  |
| Simpson et al. 2023. GRIIS United States (Contiguous) v2.0. | https://doi.org/10.5066/p9kfftod | x | x |
| Simpson et al. 2023. GRIIS Hawaii (USA) v2.0. | https://doi.org/10.5066/p9kfftod | x | x |
| Simpson et al. 2023. GRIIS Alaska (USA) v2.0. | https://doi.org/10.5066/p9kfftod | x |  |
| Pagad 2022. GRIIS Venezuela v1.2. | https://cloud.gbif.org/griis/resource?r=griis-venezuela&v=1.2 | x |  |
| Anh et al. 2020. GRIIS Vietnam v1.3. | https://cloud.gbif.org/griis/resource?r=griis-vietnam&v=1.3 | x |  |
| Graziosi et al. 2020. Plants, People, Planet. 2020;2:14–28 | https://doi.org/10.1002/ppp3.31 | x | x |
| Gryzenhout et al. 2004. Stud. Mycol. 50:119–142. |  | x | x |
| RGB Kew 2024. HerbIMI database.* | https://www.herbimi.info | x | x |
| RGB Kew 2024. Herbtrack database.* | https://herbtrack.science.kew.org/ | x | x |
| Jaeger & Charles Darwin Foundation 2019. Alien phytopathogenic fungi checklist. | <https://datazone.darwinfoundation.org/en/checklist/checklists-archive> | x | x |
| Kiss et al. 2020. Front. Microbiol. 11:1571. | https://doi.org/10.3389/fmicb.2020.01571 | x | x |
| Mulenko et al. 2010. Biol. Invasions Pol. 1:43–64. |  | x | x |
| MyCoPortal 2020. Mycology Collections Portal. | https://www.mycoportal.org/portal/index.php | x | x |
| Nahrung & Carnegie 2020. Front. For. Glob. Change 3:1–12. | https://doi.org/10.3389/ffgc.2020.00037 | x |  |
| Bufford et al. 2016. New Phytol. 212: 657-667 | https://doi.org/10.1111/nph.14077 | x | x |
| Santini et al. 2013. New Phytol. 197(1):238–250. | https://doi.org/10.1111/j.1469-8137.2012.04364.x | x | x |
| Talhinhas & Baroncelli 2021. Fungal Divers. 110:109–198. | https://doi.org/10.1007/s13225-021-00491-9 | x | x |
| Talhinhas & Baroncelli 2023. Mycosphere 14(2):158–261. | https://doi.org/10.5943/mycosphere/14/si2/4 | x | x |
| Talhinhas et al. 2019. Sydowia 71:65–84. | https://doi.org/10.12905/0380.sydowia71-2019-0065 | x | x |
| Voglmayr et al. 2023. Biol. Invasions. 25:27-38 | https://doi.org/10.1007/s10530-022-02896-2 | x | x |
| Xu & Qiang 2018. China’s Invasive Alien Species. | collected through correspondence | x | x |
| Burgess & Scott. 2023. Mendeley Data v1. | https://doi.org/10.17632/6rshgjfr8w.1 | x | x |

***Table S3 Biogeographic status of pathogens and their hosts across regions.*** *Proportion table showing percentages of observed combinations (n= 96 431 region–pathogen–host records).*

|  | | **Plant host** | | | |
| --- | --- | --- | --- | --- | --- |
|  |  | **alien** | **native** | **alien and native** | **unknown** |
| **Pathogen** | **alien/cryptogenic** | 16.4 | 7.4 | 1.5 | 1.2 |
|  | **native** | 0.1 | 0.7 | 0.0 | 0.0 |
|  | **unknown** | 29.2 | 34.2 | 5.0 | 4.2 |

***Table S4*** ***Association types in pathogens‘ alien ranges.*** *Counts and percentages by association type, with novelty indicated* *(n= 13 609 pathogen–host records).*

| **association** | **novelty** | **%** | **count** |
| --- | --- | --- | --- |
| **CO-INTRODUCTION** | FALSE | 1.13 | 154 |
| **CO-XENIC** | TRUE | 17.19 | 2339 |
| **NOVEL** | TRUE | 26.03 | 3543 |
| **NOVEL AND CO-XENIC** | TRUE | 3.50 | 476 |
| **NOVEL OR CO-XENIC** | TRUE | 6.36 | 865 |
| **CO-INTRODUCTION OR CO-XENIC** | UNKNOWN | 19.95 | 2715 |
| **UNDEFINED** | UNKNOWN | 25.84 | 3517 |

***Table S5 Top 25 plant species by pathogen richness across association types.***

| **Plant species** | **All fungal taxa** | **Plant species** | **Novel + co-xenic** | **Plant species** | **Novel only** | |
| --- | --- | --- | --- | --- | --- | --- |
| *Malus domestica* | 71 | *Citrus × aurantium* | 38 | *Quercus robur* | | 14 |
| *Pinus radiata* | 71 | *Citrus × limon* | 32 | *Quercus ilex* | | 12 |
| *Vitis vinifera* | 53 | *Malus domestica* | 18 | *Quercus petraea* | | 11 |
| *Lolium perenne* | 45 | *Persea americana* | 15 | *Agathis australis* | | 10 |
| *Pyrus communis* | 45 | *Quercus ilex* | 15 | *Quercus cerris* | | 10 |
| *Solanum lycopersicum* | 45 | *Picea abies* | 14 | *Fraxinus excelsior* | | 9 |
| *Triticum aestivum* | 44 | *Prunus avium* | 14 | *Picea abies* | | 9 |
| *Prunus persica* | 43 | *Prunus domestica* | 14 | *Quercus velutina* | | 9 |
| *Prunus avium* | 42 | *Prunus persica* | 14 | *Ulmus glabra* | | 9 |
| *Actinidia chinensis* | 40 | *Quercus robur* | 14 | *Alnus glutinosa* | | 8 |
| *Persea americana* | 40 | *Hevea brasiliensis* | 13 | *Cornus florida* | | 8 |
| *Zea mays* | 40 | *Pinus radiata* | 13 | *Eucalyptus marginata* | | 8 |
| *Hordeum vulgare* | 39 | *Solanum lycopersicum* | 13 | *Quercus agrifolia* | | 8 |
| *Citrus × aurantium* | 38 | *Solanum tuberosum* | 13 | *Quercus pubescens* | | 8 |
| *Malus sylvestris* | 38 | *Vitis vinifera* | 13 | *Alnus rubra* | | 7 |
| *Medicago sativa* | 37 | *Chamaecyparis lawsoniana* | 12 | *Arbutus unedo* | | 7 |
| *Prunus armeniaca* | 35 | *Pseudotsuga menziesii* | 12 | *Chamelaucium uncinatum* | | 7 |
| *Solanum tuberosum* | 34 | *Quercus alba* | 12 | *Juniperus phoenicea* | | 7 |
| *Eucalyptus globulus* | 33 | *Agathis australis* | 11 | *Myrtus communis* | | 7 |
| *Citrus × limon* | 32 | *Fraxinus excelsior* | 11 | *Nothofagus fusca* | | 7 |
| *Capsicum annuum* | 31 | *Pinus strobus* | 11 | *Ulmus minor* | | 7 |
| *Chamaecyparis lawsoniana* | 31 | *Quercus cerris* | 11 | *Agonis flexuosa* | | 6 |
| *Castanea sativa* | 30 | *Quercus petraea* | 11 | *Araucaria cunninghamii* | | 6 |
| *Dianthus caryophyllus* | 30 | *Quercus pyrenaica* | 11 | *Banksia attenuata* | | 6 |
| *Pseudotsuga menziesii* | 30 | *Quercus rubra* | 11 | *Banksia prionotes* | | 6 |

***Table S6*** ***Clustered versus dispersed observed host phylogenetic host ranges across fungal classes.*** *Counts and percentages of taxa with at least two recorded hosts showing significantly clustered or dispersed observed phylogenetic host range (ses.MPD_obs_) across 16 fungal classes plus pooled incertae sedis (Ascomycota). Total n = 1117.*

| **Phylum** | **Class** | **cluster** | **%** | **dispersed** | **%** | **all** |
| --- | --- | --- | --- | --- | --- | --- |
| Ascomycota | Dothideomycetes | 360 | 94.0 | 23 | 6.0 | 383 |
| Ascomycota | Eurotiomycetes | 1 | 100.0 |  |  | 1 |
| Ascomycota | *Incertae sedis* | 8 | 100.0 |  |  | 8 |
| Ascomycota | Leotiomycetes | 167 | 97.1 | 5 | 2.9 | 172 |
| Ascomycota | Saccharomycetes | 1 | 100.0 |  |  | 1 |
| Ascomycota | Sordariomycetes | 140 | 76.5 | 43 | 23.5 | 183 |
| Ascomycota | Taphrinomycetes | 11 | 100.0 |  |  | 11 |
| Basidiomycota | Agaricomycetes | 6 | 37.5 | 10 | 62.5 | 16 |
| Basidiomycota | Entorrhizomycetes | 1 | 100.0 |  |  | 1 |
| Basidiomycota | Exobasidiomycetes | 30 | 100.0 |  |  | 30 |
| Basidiomycota | Microbotryomycetes | 6 | 100.0 |  |  | 6 |
| Basidiomycota | Pucciniomycetes | 155 | 96.3 | 6 | 3.7 | 161 |
| Basidiomycota | Ustilaginomycetes | 36 | 100.0 |  |  | 36 |
| Blastocladiomycota | Blastocladiomycetes | 3 | 100.0 |  |  | 3 |
| Chytridiomycota | Chytridiomycetes | 7 | 100.0 |  |  | 7 |
| Oomycota | Peronosporomycetes | 54 | 59.3 | 37 | 40.7 | 91 |
| Oomycota | Saprolegniomycetes | 7 | 100.0 |  |  | 7 |

***Table S7 The 15 taxa with broadest observed host ranges.*** *Taxa are ranked by SR, ses.MPD and PD respectively. Taxa with high species richness and phylogenetic host range breadth, i.e. appearing in both, the SR and ses.MPD listing, are highlighted in bold red. PD is shown for completeness. Phyla: A = Ascomycota, B = Basidiomycota, O = Oomycota* ***.***

| **Phylum** | **Class** | **Taxon name** | **SR** | **Phylum** | **Class** | **Taxon name** | **ses.MPD** | **Phylum** | **Class** | **Taxon name** | **PD** |
| --- | --- | --- | --- | --- | --- | --- | --- | --- | --- | --- | --- |
| O | Peronosporo | ***Phytophthora cinnamomi*** | 895 | B | Agarico | ***Armillaria mellea*** | 55.49 | O | Peronosporo | *Phytophthora cinnamomi* | 26 347.32 |
| B | Agarico | *Athelia rolfsii* | 738 | O | Peronosporo | ***Phytophthora cinnamomi*** | 35.42 | A | Dothideo | *Lasiodiplodia theobromae* | 25 143.38 |
| A | Dothideo | *Stemphylium vesicarium* | 736 | A | Sordario | *Pesotum piceae* | 30.91 | B | Agarico | *Athelia rolfsii* | 24 921.23 |
| A | Dothideo | *Lasiodiplodia theobromae* | 671 | O | Peronosporo | *Globisporangium ultimum* | 28.84 | A | Dothideo | *Stemphylium vesicarium* | 22 757.90 |
| B | Agarico | ***Armillaria mellea*** | 614 | B | Agarico | ***Ganoderma applanatum*** | 28.45 | O | Peronosporo | *Phytophthora nicotianae* | 21 779.91 |
| O | Peronosporo | *Phytophthora nicotianae* | 563 | A | Dothideo | ***Macrophomina phaseolina*** | 27.12 | B | Agarico | *Armillaria mellea* | 20 349.87 |
| A | Leotio | *Sclerotinia sclerotiorum* | 530 | O | Peronosporo | ***Phytophthora cactorum*** | 26.97 | A | Leotio | *Sclerotinia sclerotiorum* | 16 812.35 |
| A | Leotio | *Phyllactinia guttata* | 526 | O | Peronosporo | ***Phytophthora hedraiandra*** | 24.76 | A | Dothideo | *Macrophomina phaseolina* | 16 505.55 |
| A | Dothideo | ***Macrophomina phaseolina*** | 449 | O | Peronosporo | *Phytophthora citrophthora* | 24.14 | A | Dothideo | *Botryosphaeria dothidea* | 16 115.81 |
| O | Peronosporo | ***Phytophthora cactorum*** | 431 | A | Dothideo | *Epicoccum nigrum* | 22.81 | O | Peronosporo | *Phytophthora cactorum* | 15 955.15 |
| A | Dothideo | *Botryosphaeria dothidea* | 430 | O | Peronosporo | ***Phytophthora cryptogea*** | 21.96 | A | Leotio | *Phyllactinia guttata* | 15 836.74 |
| A | Sordario | *Nigrospora oryzae* | 390 | O | Peronosporo | *Globisporangium irregulare* | 20.94 | A | Dothideo | *Epicoccum nigrum* | 15 569.51 |
| O | Peronosporo | ***Phytophthora cryptogea*** | 384 | A | Dothideo | *Neofusicoccum parvum* | 19.63 | A | Sordario | *Nigrospora oryzae* | 15 462.04 |
| B | Agarico | ***Ganoderma applanatum*** | 355 | B | Agarico | *Rigidoporus vinctus* | 19.24 | O | Peronosporo | *Phytophthora cryptogea* | 15 405.34 |
| O | Peronosporo | ***Phytophthora hedraiandra*** | 355 | A | Dothideo | *Botryosphaeria stevensii* | 18.52 | O | Peronosporo | *Phytophthora hedraiandra* | 14 342.42 |

***Table S8*** ***The 15 taxa with phylogenetically most constrained observed host ranges (lowest ses.MPD_obs_)****. Phyla: A = Ascomycota, B = Basidiomycota, O = Oomycota.*

| **P** | **Class** | **Family** | **Taxon name** | **ses.MPD** | **SR** |
| --- | --- | --- | --- | --- | --- |
| B | Pucciniomycetes | Pucciniaceae | *Puccinia hieracii* | -67.15 | 302 |
| B | Pucciniomycetes | Pucciniaceae | *Puccinia calcitrapae* | -56.43 | 230 |
| B | Pucciniomycetes | Sphaerophragmiaceae | *Austropuccinia psidii* | -55.63 | 293 |
| A | Leotiomycetes | Erysiphaceae | *Erysiphe heraclei* | -53.88 | 198 |
| B | Pucciniomycetes | Pucciniaceae | *Puccinia brachypodii* | -48.66 | 204 |
| A | Dothideomycetes | Mycosphaerellaceae | *Graminopassalora graminis* | -47.64 | 181 |
| B | Pucciniomycetes | Melampsoraceae | *Melampsora euphorbiae* | -47.56 | 157 |
| A | Leotiomycetes | Erysiphaceae | *Neoerysiphe galeopsidis* | -46.13 | 211 |
| O | Peronosporomycetes | Peronosporaceae | *Phytophthora infestans* | -41.99 | 148 |
| B | Pucciniomycetes | Pucciniaceae | *Puccinia violae* | -40.89 | 125 |
| A | Leotiomycetes | Erysiphaceae | *Erysiphe trifoliorum* | -40.19 | 187 |
| B | Pucciniomycetes | Pucciniaceae | *Puccinia menthae* | -39.63 | 116 |
| B | Pucciniomycetes | Pucciniaceae | *Puccinia malvacearum* | -37.68 | 80 |
| A | Leotiomycetes | Erysiphaceae | *Golovinomyces asterum* | -35.98 | 72 |
| B | Pucciniomycetes | Pileolariaceae | *Uromycladium tepperianum* | -35.93 | 122 |

***Table S9*** ***Summary of the binomial generalised linear mixed model.*** *The model (family: binomial, link: logit) was fit by maximum likelihood (Laplace approximation, optimizer: optimx). Number of observations: 24 622. Groups: 201 regions, 375 fungal genera, 1630 plant genera. SD of random effects (intercept only): region = 0.74, pathogen genus = 1.96, plant genus = 2.58. Marginal Pseudo-R²: 0.18. Conditional Pseudo-R²: 0.81.*

| **group** | **Fixed effects** | **Estimate** | **Std. Error** | **z value** | **p** |
| --- | --- | --- | --- | --- | --- |
|  | Intercept | 1.31 | 0.22 | 6.09 | <0.0001 |
| **Region** | **SR_PLANT COMMUNITY_** | **0.17** | **0.07** | **2.56** | **0.0104** |
|  | Artificial areas | 0.02 | 0.04 | 0.45 | 0.6533 |
|  | Food production areas | -0.04 | 0.04 | -1.00 | 0.3188 |
| **Pathogen x region** | **bmntd_PLANT COMMUNITY x HOST COMMUNITY_** | **-0.14** | **0.05** | **-2.93** | **0.0034** |
|  | **Economically used hosts in region** | **0.53** | **0.06** | **8.66** | **<0.0001** |
| **Pathogen** | **SR_restr_** | **-0.69** | **0.06** | **-10.91** | **<0.0001** |
|  | **ses.MPD_restr_** | **0.27** | **0.06** | **4.51** | **<0.0001** |
|  | **Alien distribution** | **1.26** | **0.06** | **21.70** | **<0.0001** |
| **Plant** | **Economic use** | **-0.71** | **0.07** | **-9.85** | **<0.0001** |
|  | Woody | -0.05 | 0.12 | -0.45 | 0.6552 |
|  | **Native distribution** | **-0.67** | **0.03** | **-20.19** | **<0.0001** |
|  | SR_FAMILY_ | 0.12 | 0.08 | 1.51 | 0.1319 |
|  | SR_GENUS_ | -0.02 | 0.12 | -0.19 | 0.8471 |

# **Methods S1** Additional information on data sources and data preparation.

## Global Database of Alien Pathogenic Fungi

### **Distribution ranges**

We first exported all unique pathogen-region combinations in the database and standardised them to a common set of spatial units (Fig. S2) to ensure comparability among pathogens when calculating metrics such as alien range. TDWG Level 3 regions that included two or few more countries (e.g. due to former political borders) were split up, so that each country was represented and the study resolution met. This concerned: Borneo, Czechoslovakia, Gulf States, Korea, New Guinea, Sudan, Yugoslavia. We excluded those records that did clearly exceed the study resolution (i.e. TDWG Level 2 regions; Caribbean, Caucasus, Central America, Indian Subcontinent, Malesia, Northern Europe, Southern Africa, Southern South America, Western Asia, as well as USSR). If available, we included information on the pathogens biogeographic status (note that the database also contains distribution data from general-purpose databases, that do not necessarily come with such information).

### **Associations globally and on regional level**

Association records were intended to maximize information for assessing host range breadth and classification of association types. Therefore, to characterize the overall host range breadth of the included alien fungal pathogens we first exported all available host association data, regardless of whether this information was coupled to a specific standardised region. This dataset thus included regional (= study resolution), supraregional (= exceeding study resolution) and global records (= no spatial information). Genus-level host plant records were discarded. Associations that were only supported by questionable records (e.g. host name flagged with a question mark), were discarded. Supraregional records were only retained when no equivalent regional records were available. To avoid duplication, supraregional records that were fully represented by nested lower-level records were excluded from subsequent analyses. In total 1371 supraregional records records were included, encompassing 373 taxa and six regions (Caucasus, Central America, Indian Subcontinent, Southern Africa, Southern South America and USSR).

#### *Validation/Correction of associations*

To account for significant changes in the taxonomy and systematics of fungal taxa, we integrated expert feedback, e.g. from Uwe Braun for the *Mycosphaerellaceae* and *Erysiphaceae*. Further, if available, we included curated datasets that use up-to-date taxonomy and sensu stricto concepts for taxa, e.g. the datasets of Talhinhas & Baroncelli (2021, 2023) for *Colletotrichum*, or the dataset of Batista *et al.* (2021) for *Botryosphaeriaceae*. Those were treated as authority sources. The corrections made were either

- taxonomy-based: exclusion of taxa, updating of scientific names or marking as *sensu lato*, see Table S1
- host association-based: exclusion of records, correction of the assigned pathogen or host name, marking as sensu lato/stricto
- region based: exclusion of records from specific regions or updating biogeographic status, such as classifying *Erysiphaceae* as alien in Australia, see (Kiss *et al.*, 2020)
- reference-based: for example, Talhinhas & Baroncelli (2021, 2023) for *Colletotrichum* (marking those records as reliable and based on a *sensu stricto* species concept)
- association-type based: (i.e. co-introduced pathogens will be alien in the alien range of the host, therefore gaps in status data can be filled. This can be applied to fungal taxa that are known to be monophagous on a specific host or geographically restricted genus)

The following corrections were implemented:

- exclude the whole fungal taxon
- flag as sensu stricto or sensu lato
- exclude a specific record
- correct the biogeographic status of the taxon
- correct the name of the taxon
- correct the association type of a pathogen-host pair

If name corrections were applied to records, the biogeographic status was changed to ‘unknown’. An exception was made for records that also had a status correction, as this step was implemented after name corrections, and therefore the status was accurate for the new name.

##### **Biogeographic status of the pathogens**

The biogeographic status of the pathogen was either derived from the utilized source or from native range comparison. For each record the status was harmonized to either “native”, “unknown” or “alien/cryptogenic”. After aggregation of the raw dataset to region-pathogen, the status was again compared. If multiple statuses were found for one combination it was harmonized hierarchically: “alien” > “native” > “unknown”.

In a second step, native range information for pathogens was compiled from literature (see lookup_native_ranges.xlsx). We adopted a conservative approach, rather assigning broader potential native ranges (e.g. “Eurasia,” “Northern Hemisphere”), or multiple continents when sources conflicted, to avoid inflating alien records. For each pathogen, native ranges were standardized, using continental (Level 1) or subcontinental (Level 2) TDWG region names (Brummitt, 2001).

Record-level checks primarily compared occurrences against Level 1 native ranges. Where Level 2 information was available, it was used to refine matches, but alien status assignment remained conservative and relied mainly on broader continental ranges. A binary flag was assigned to indicate occurrences outside the native range. Pathogen records with “unknown” biogeographic status and a mismatch were marked as alien. We did not assign native status where occurrences fell within the assigned native ranges, as we deemed this delineation not precise enough to make statements about status within those (sub)continental regions. While many records remain classified as “unknown,” this reflects both current knowledge gaps and our conservative strategy.

##### **Phylogenetic matching of plant species names**

If a plant host species was not represented in the plant phylogeny, but its genus was, then the species was added to the genus root (see “Plant taxonomy and phylogeny”). 21 plant species were discarded because the according genus was not represented in the tree (0.1% of the plant taxa, amounting to 60 region-pathogen-host records; 0.03%).

#### *Genus level plant records*

Sometimes pathogen-host association information was given on genus level only. Some of those were not represented in the phylogeny and would have been discarded anyways. Others were assumed to be redundant as the occurrence of the fungal pathogen on the plant genus was already covered by a more precise species-level record. Note that we cannot exclude the possibility, that the genus-level record actually referred to another plant species of the genus. On the other hand, genus-level might be too coarse as taxonomic resolution especially for narrow-host-range pathogens.

Finally, remaining 10 299 region-pathogen-plant genera records are not represented by a plant species record in the dataset (concerned 941 fungal pathogen taxa and 1126 plant genera in the final datasets).
Fungal pathogens that were only represented with genus-level host records were excluded from the analyses.

## Plant taxonomy and phylogeny

For this study we used WCVP V.14 (28 May 2025) and cross-checked all plant names with the snapshot provided by the R package ‘rWCVPdata’ (Govaerts *et al.*, 2021; Govaerts, 2025). Names were matched using exact and fuzzy matching, and names with a match similarity smaller than 0.8 were excluded.

The tree of Smith & Brown (2018) combines genetic data from public repositories with phylogenetic data and is based on a hierarchical clustering analysis. Note that this tree contains polytomies, i.e., multifurcations with three or more children. The modified tree Yang *et al.* (2021) includes additional taxa that were added to their genus root if any of their congeneric taxa was included or to their family root if any taxon of the same family is included respectively, resulting in additional polytomies. Note that we used the version of the tree based on WCVP V9 (Yang, pers. comm.) and updated the taxonomy to WCVP V14, by reconciling identifiers and accepted names.

We added 303 additional plant taxa from our associations, which were previously not represented in the phylogeny dataset to the corresponding genus root (29 species were discarded due to missing genus representation), using the code provided by Q. Yang. Many of those taxa are hybrids (~45%), for example, artificial hybrids of cultivated plants, and/or from plant genera popular in horticulture or agriculture and forestry (e.g. *Viburnum, Citrus, Magnolia, Rhododendron, Begonia, Syringa, Rubus, Prunus…*). For polytypic genera, taxa were inserted at the most recent common ancestor (MRCA) of existing congeners when possible, whereas for monospecific genera they were attached to the parent node of the single representative. We did not treat hybrids differently when adding them; they were attached to the genus root. We acknowledge that this approach may introduce additional uncertainty into the phylogeny, but also allows retention of these taxa while maintaining comparability with the underlying framework.

## Plant distribution

### **GIFT**

**GIFT (Global Inventory of Floras and Traits) is a database that provides plant checklists worldwide, as well as trait and phylogenetic information (https://gift.uni-goettingen.de/home).**

#### *Version*

This study utilized GIFT V.3.2 (June 13 2024), which is the most recent stable snapshot of the database. This version contains a significant update by integrating distribution data and the taxonomic standard of WCVP (Govaerts *et al.*, 2021).

#### *Citation*

**Weigelt P, König C, Kreft H. 2020.** GIFT – A Global Inventory of Floras and Traits for macroecology and biogeography. *J Biogeogr* **47**: 16– 43. <https://doi.org/10.1111/jbi.13623>

**Weigelt P, Denelle P. 2024.** GIFT: Access to the Global Inventory of Floras and Traits (GIFT). R package version 1.3.2, <https://CRAN.R-project.org/package=GIFT>

**Denelle P, Weigelt P, Kreft H. 2023.** GIFT – An R package to access the Global Inventory of Floras and Traits. *Methods Ecol Evol* **14**: 2738-2748. <https://doi-org/10.1111/2041-210X.14213>

#### *Export*

Regional plant lists were downloaded from on June 4 2025. We retrieved checklists for Spermatophyta, including all biogeographic status categories (native, endemic, alien), by using spatial queries based on our study regions. For each feature, we retrieved relevant plant lists by using the overlap-constraint “centroid_inside” (i.e. GIFT data from regions where the centroid lies inside the feature were included). For islands lacking results, we used the more liberal overlap-constraint “shape_intersect”. We manually checked the retrieved data and excluded lists from regions that were exceeding the target resolution (e.g., sub/continental scale, custom multi-country polygons, etc.).

Excluded lists:

- Spatial extent is not matching our study resolution*:*

*“Caribbean”, “Indo-China”, “Andaman & Nicobar”, “East Tropical Africa”, “South China Sea”, “Southwest Caribbean”, “Sri Lanka and Maledive”, “Tropical West Africa”, “West Indies excl. Trinidad and Tobago”, " Yugoslavia”, “Serbia & Kosovo”, “Madeira and Selvagens”,* …

To keep the workflow consistent, we used only the spatial query to derive regional plant information from GIFT. Unlike in WCVP, we did not split former political regions such as Czechoslovakia. However, we checked all region names (i.e. TDWG lvl3) that were split in the WCVP workflow; in GIFT this concerned only six lists (Sudan, Malaya, North Caucasus, Lebanon-Syria, Yugoslavia, Baltic States). All of these were already covered by one or more other GIFT lists, thus integrated via spatial matching.

- Overlaps with specific study features do not match their extent:
- Remove *“India” for Andaman Islands and Nicobar Islands.*
- Remove *“Newfoundland” from “Saint Pierre and Miquelon”* (French Islands, c. 25km in front of Newfoundland)
- *“Santa Cruz Islands”* as separate entity and *“Solomon islands excluding Santa Cruz Islands”*

#### *Taxonomic standardisation*

GIFT V.3.2 standardizes taxon names primarily according to WCVP V9 (Denelle *et al.*, 2023). We cross-checked all names against a WCVP V14 snapshot to update taxonomy where necessary. Unplaced/not accepted names and names above species level were discarded, and infraspecific ranks were standardized to species level.

#### *Spatial aggregation*

Overlapping GIFT regions that were assigned to the same feature of our study resolution were aggregated accordingly.

#### *Biogeographic status*

As GIFT contains native (incl. endemic) and alien (incl. naturalized) occurrences of species, the taxonomic standardisation and spatial aggregation may lead to conflicting biogeographic status trough differing classifications. Those conflicts can arise through taxonomic standardisation of alien subspecies/varieties in the native region of a species or spatial aggregation, if a species is alien in one part of a country and native in another part (e.g., a species native to the US East Coast introduced to the US West Coast) (P. Weigelt, pers. communication). For such within-database conflicts of native-alien status (2.3 % of plant-region records), we used the classification ‘native and alien’. We did not distinguish between native and endemic occurrences. Further we removed all biogeographic status classifications that were marked as questionable, which – in part – contributed to 2.7% of plant species - region records lacking an assigned biogeographic status, i.e. ’unknown’.

#### *Final numbers*

After taxonomic and spatial standardisation and aggregation the prepared dataset consisted of 973 566 unique plant species-region records for 260 regions and 281 678 plant species names. Of those 87.2% are considered native, 7.8% alien, 2.3% are considered native and alien and 2.7% lack information on biogeographic status (i.e. unknown status).

### **GloNAF**

#### The GloNAF (Global Naturalized Alien Flora) database is a continuously updated compilation of alien naturalized vascular plant inventories for geographic regions globally.

#### *Version*

This study utilized GloNAF V.2.02, which is a snapshot of the database last updated on February 24, 2025.

#### *Citation*

#### Zenodo Dataset (last updated February 25 2025):

#### Davis AJS, Dawson W, Essl F, Kreft H, Lenzner B, Pergl J, Pyšek P, Weigelt P, Winter M, van Kleunen M. 2025. Global Naturalized Alien Flora (GloNAF). Open access data to support research on understanding global plant invasions. [Data set]. Zenodo. https://doi.org/10.5281/zenodo.14696776

#### GloNAF publication:

**van Kleunen M, Pyšek P, Dawson W, Essl F, Kreft H, Pergl J, Weigelt P, Stein A, Dullinger S, König C, *et al.*** **2019**. The Global Naturalized Alien Flora (GloNAF) database. *Ecology* 100: e02542.

#### *Export*

All necessary datasets were downloaded from the Zenodo repository, namely the tables glonaf_flora2, glonaf_region, glonaf_taxon_wcvp, and the regions shapefile. The initial distribution dataset contained 349 271 observations for 60 757 original input taxon names across 1343 regions.

#### *Taxonomic standardisation*

GloNAF V.2.02 uses WCVP V12 (Govaerts *et al.*, 2021) for taxonomic standardisation. We cross-checked the ‘*accepted_plant_name_id’* column in the table glonaf_taxon against a WCVP V14 snapshot to update taxonomy where necessary. Synonyms were resolved and only accepted names kept (taxon status is ‘accepted’ or ‘artificial hybrid’). Taxa that did not match with an accepted name (2057 taxa) and non-target taxa, i.e. those that do not belong to the Spermatophyta (1258 taxa) were excluded. This resulted in 19 651 standardized accepted species names before spatial aggregation.

#### *Spatial aggregation*

To match the regions used by GloNAF to our study resolution we intersected both spatial datasets. Regions in GloNAF that showed an overlap of at least 90% and/or a precise name matching or a match in the region translation table were attributed to the according study region (1430 of the GloNAF regions). Regions exceeding the target distribution were discarded.

Type of matching:

- Completely within region: 295 features
- Nearly within (at least 90% overlap): 1104 features
- Partial overlap (<90%) but exact name matching: 30 features (e.g. due to political border conflicts, islands depicted in varying detail etc.)

#### *Biogeographic status*

As GloNAF only contains alien occurrence records, no conflicts in the biogeographic status occurred after taxonomic standardisation and spatial aggregation. While GloNAF contains information on different stages of invasion, we did not differentiate between naturalized and invasive occurrences.

#### *Final numbers*

After taxonomic and spatial standardisation and aggregation the prepared dataset consisted of 76 405 unique alien plant species-region records for 235 regions and 14 262 plant species names.

### **The World Checklist of Vascular Plants**

#### The World Checklist of Vascular Plants (WCVP) is a continuously updated list of plant species and their distributions, providing regional checklists and expert reviewed taxonomic data for all vascular plants. The WCVP taxonomic and distribution data is accessed via the package rWCVPdata (Govaerts et al., 2021; Govaerts, 2025).

#### *Version*

This study utilized WCVP V.14, which is a snapshot of the database last updated on May 28, 2025.

#### *Citation*

**Govaerts R (ed.). 2025**. WCVP: World Checklist of Vascular Plants. Facilitated by the Royal Botanic Gardens, Kew. <https://doi.org/10.34885/nswv-8994> [v14 accessed 28 May 2025].

**Govaerts R, Lughadha NE, Black N, Turner R, Paton A. 2021.** The World Checklist of Vascular Plants, a continuously updated resource for exploring global plant diversity. *Sci Data* **8**(1):215. <https://doi.org/10.1038/s41597-021-00997-6>

#### *Export*

Distribution data for all accepted species-level names was derived via rWCVPData (Govaerts *et al.*, 2021; Govaerts, 2025). rWCVPData redistributes snapshots of the WCVP database. The full dataset included 1 646 406 records for 428 944 species or infra-species names.

#### *Taxonomic standardisation*

We filtered for accepted species level names (i.e., taxon rank is ‘Species’ and taxon status is ‘accepted’ or ‘artificial hybrid’) and excluded non-target species, i.e. those that do not belong to the Spermatophyta (13 689). This resulted in 349 619 standardized accepted species names.

#### *Spatial aggregation*

We matched the records to our study resolution using the TDWG region codes. While most records could be matched directly, some regions exceed the spatial resolution used in our study. To avoid larger gaps in data coverage, Level 3 regions that included two or a few more countries (due to former political borders etc.) were subdivided, so that individual regions could be represented and the study resolution met. This applied to the following regions: Baltic States, Borneo, Czechia-Slovakia, East Himalaya, Gulf States, Korea, Lebanon-Syria, Malaya, New Guinea, North Caucasus, Sudan, Transcaucasus, West Himalaya, and NW. Balkan Pen. Higher level regions and non-matching LVL3 archipelagos (i.e. Central American Pacific Islands, Gulf of Guinea Islands, Marianas, South China Sea, Southwest Caribbean) were removed. The resulting 365 regions were harmonised to the study resolution, resulting in a 99.7% match success.

#### *Biogeographic status*

Records that were labelled ‘extinct’ or ‘location doubtful’ were discarded. The prepared WCVP dataset contained 90.9% native plant species-region records, 8.8% alien records and 0.3% of records that through spatial aggregation were considered both, native and alien. This can happen when spatial aggregation combined regions in which a species differed in biogeographic status, for example between mainland and island territories of a country (e.g. Eastern and Western United States, Spain and Baleares). For those native/alien conflicts the status was defined as ‘native and alien’.

#### *Final numbers*

After taxonomic and spatial standardisation and aggregation the prepared dataset consisted of 1 064 336 plant species-region records for 241 regions and 349 138 standardized plant species.

### **Combining the sources**

The prepared database exports were combined, and the standardised plant names were matched to the phylogenetic tree. Names that did not match (4.5% of species names, corresponding to 1% of records) were removed from the dataset. While GIFT contributed most of the records, the overlap between databases was considerable: 52.5% of plant species-region combinations were shared between GIFT and WCVP, 25.4% were unique to WCVP, 17.2% to GIFT, 1.9% found in all three databases, 1% were unique to GloNAF, and 1.1 % were shared between GloNAF and WCVP. As each database contributed unique records, all sources were retained to maximise the amount of available data on regional plant species pools.

#### Harmonizing biogeographic status - Between database conflicts

Regions in WCVP, GIFT and GloNAF vary considerably in size, ranging from small islands or protected areas to subcontinental regions and may overlap or be nested. Aggregating such records to a common spatial extent can introduce conflicts due in biogeographic status among aggregated plant-region records. Likewise, the taxonomic standardisation of synonyms or subspecies/varieties to species level can lead to differing status (Weigelt, pers. comm.).

We harmonized the combined data, by resolving inconsistencies in biogeographic status across plant-region records, using the following workflow. Because GIFT inherits status assignments from heterogeneous original sources, which may differ in definitions or contain misclassifications, we first flagged records based solely on GIFT classified as ‘native’ or ‘native and alien’ but occurred outside its native continental region (TDWG Level 1) according to WCVP, and where no directly neighbouring region belonged to the same native continental range. In a subsequent step, we compared all status information available across the three databases and the flags and resolved inconsistencies using a harmonisation scheme covering 34 possible status combination scenarios (Table 1).

Table 1. Potential combinations of biogeographic status for a plant-region record (‘native’, ‘alien’, ‘native and alien’) across the three databases.

| # | Status GIFT conflict | Status GIFT | Status GloNAF | Status WCVP | Frequency | Harmonized status | Conflict | Uncert-ainty | Scenario Type |
| --- | --- | --- | --- | --- | --- | --- | --- | --- | --- |
| 1 |  | native |  | native | 644 474 | **native** |  |  | Consistent native |
| 2 |  |  |  | native | 280 443 | **native** |  |  | Consistent native |
| 3 |  | native |  |  | 155 444 | **native** |  |  | Consistent native |
| 4 |  |  |  | alien | 30 617 | **alien** |  |  | Consistent alien |
| 5 | TRUE (indirect) | native |  |  | 30 157 | **alien** | TRUE |  | Conflict |
| 6 |  | alien | alien | alien | 23 215 | **alien** |  |  | Consistent alien |
| 7 |  | alien |  |  | 22 554 | **alien** |  |  | Consistent alien |
| 8 |  | alien |  | alien | 17 364 | **alien** |  |  | Consistent alien |
| 9 |  |  | alien |  | 16 492 | **alien** |  |  | Consistent alien |
| 10 |  | alien and native |  | native | 8555 | **native** |  | TRUE | Uncertain |
| 11 |  |  | alien | alien | 8438 | **alien** |  |  | Consistent alien |
| 12 |  | alien | alien |  | 7718 | **alien** |  |  | Consistent alien |
| 13 |  |  |  |  | 7030 | **unknown** |  |  | Unknown |
| 14 | TRUE (direct) | native |  | alien | 6453 | **alien** | TRUE |  | Conflict |
| 15 |  | native | alien | native | 5405 | **native** | TRUE |  | Conflict |
| 16 |  | alien and native |  | alien | 3373 | **alien** |  | TRUE | Uncertain |
| 17 |  | alien and native | alien | alien | 3207 | **alien** |  | TRUE | Uncertain |
| 18 |  | alien |  | native | 3007 | **native** | TRUE |  | Conflict |
| 19 |  | alien and native |  |  | 2910 | **native and alien** |  | TRUE | Uncertain |
| 20 |  | alien and native | alien | native | 2722 | **native and alien** | TRUE | TRUE | Conflict+  Uncertain |
| 21 | TRUE (direct) | native | alien | alien | 1983 | **alien** | TRUE |  | Conflict |
| 22 |  | alien | alien | native | 1832 | **native** | TRUE |  | Conflict |
| 23 |  |  | alien | native | 1575 | **native** | TRUE |  | Conflict |
| 24 |  | alien and native | alien | alien and native | 826 | **native and alien** |  | TRUE | Uncertain |
| 25 | TRUE (indirect) | native | alien |  | 678 | **alien** | TRUE |  | Conflict |
| 26 | TRUE (direct) | native | alien | alien and native | 676 | **native and alien** | TRUE | TRUE | Conflict+  Uncertain |
| 27 | TRUE (direct) | native |  | alien and native | 671 | **native** |  | TRUE | Uncertain |
| 28 |  | native | alien |  | 613 | **native** | TRUE |  | Conflict |
| 29 |  | alien and native | alien |  | 516 | **native and alien** |  | TRUE | Uncertain |
| 30 |  | alien and native |  | alien and native | 301 | **native and alien** |  | TRUE | Uncertain |
| 31 |  |  |  | alien and native | 196 | **native and alien** |  | TRUE | Uncertain |
| 32 |  | alien | alien | alien and native | 110 | **native and alien** |  | TRUE | Uncertain |
| 33 |  |  | alien | alien and native | 80 | **native and alien** |  | TRUE | Uncertain |
| 34 |  | alien |  | alien and native | 68 | **native and alien** |  | TRUE | Uncertain |

***Final numbers***

The final dataset on regional plant species pools contained 1 285 830 unique plant-region records across 264 regions and 334 568 taxa. Of those records, 13.0% were classified as alien 0.7% as native and alien in a region and the remaining 85.8% as native while 0.5% lack any information on biogeographic status (i.e. unknown status).

## Modelling

### **Weights**

To account for unequal sampling effort across regions, pathogen taxa, and host plants, we applied inverse-frequency weights (1/n per category), so that frequently sampled entities received lower weights and rarely sampled entities higher weights. Weights for regions, fungi, and plants were mean-standardised and combined using their geometric mean; the resulting weights were again normalised to a mean of 1.

Because the resulting weight distribution was strongly right-skewed, we applied winsorisation (capping) to limit extreme upper values. Three upper quantile thresholds were tested (99.9%, 99.5%, 99%), with values above the threshold set to the respective quantile and weights re-normalised. The medium threshold was used for the main model and the upper and lower threshold for the sensitivity analysis. Lower weights (i.e. overrepresented entities) were unaffected. Across all weighting schemes, the most strongly downweighted entities were:

- Regions: New Zealand, United States, Australia, Great Britain and Italy, i.e. large countries and/or countries with high research effort.
- Pathogens: *Phytophthora nicotianae*, *P. cinnamomi*, *P. infestans*, *Verticillium dahliae*, *P. palmivora*, i.e. widespread and/or generalist pathogens.
- Host plants: *Solanum lycopersicum*, *Zea mays*, *Vitis vinifera*, *Malus domestica*, *Prunus persica,* i.e. agricultural crops and trees.

### **Random-effects structure**

We assessed the distribution of observations across candidate random-effect levels (region and plant/fungal taxonomic ranks), considering the recommendation to restrict complexity while ensuring adequate levels per random effect (Bolker *et al.* 2009). While replication was sufficient for the majority of regions, singleton groups were common at the species level but less frequent at higher taxonomic levels. This supported the use of genus-level random effects to reduce overfitting associated with sparsely sampled species. Higher taxonomic-level random effects (plant order and pathogen class) were tested in the sensitivity analyses. All random effects were specified as intercept-only.

### **Sensitivity analyses**

We assessed robustness of model estimates to (i) alternative weighting and random-effect structures and (ii) removal of highly influential pathogen taxa:

(i) **Alternative model specifications**

Across alternative model formulations (uncapped, capped weights at 99.9% and 99%, alternative random-effect structure), effect directions and significance were largely consistent (Supplementary Information Fig. S7). Capping thresholds did not alter inference. Excluding weights or using higher-level random effects resulted in only minor deviations.

Under alternative higher-level random-effect structures, those were limited to host taxonomic richness variables (p_fam_SR, p_genus_SR) and plant woodiness (p_woody). These host variables showed attenuation or sign changes indicating non-robust effects. The same was true for regional plant richness in the unweighted model which became non-significant; however, regional plant richness effect size is small and not strongly significant in the other models as well.

In contrast, key predictors such as bmntd between regional and host community, the occurrence of other economically used hosts, pathogen alien range size, pathogen host richness, and phylogenetic host breadth, as well as plant host economic use and native range size —remained stable in direction and significance. Overall, the sensitivity analysis confirms that conclusions made are robust to weighting and model specification.

(ii) **Influential pathogen taxa**

To test whether results were driven by a small number of dominant taxa, we excluded (1) the top 1% of pathogens (2 taxa: *Phytophthora nicotianae*, *Phy. cinnamomi*; 9.85% of observations) and (2) the top 2.5% (4 taxa: *Phytophthora nicotianae*, *Phy. cinnamomi, Botryosphaeria dothidea*, *Phy. palmivora;* 13.49% of observations).

Across both scenarios, effect directions and size remained highly consistent with the main model (Supplementary Information Fig. S8). Confidence intervals strongly overlapped, and no predictor changed its significance class or direction. Overall, model results were robust to the exclusion of highly influential taxa, indicating that results are not driven by a subset of dominant pathogens.

## References

**Batista E, Lopes A, Alves A**. **2021**. What do we know about Botryosphaeriaceae? An overview of a worldwide cured dataset. *Forests* **12**.

**Bolker BM, Brooks ME, Clark CJ, Geange SW, Poulsen JR, Stevens MHH, White JSS. 2009.** Generalized linear mixed models: a practical guide for ecology and evolution. Trends in Ecology and Evolution **24**: 127–135.

**Brummitt RK**. **2001**. *World Geographical Scheme for Recording Plant Distributions Edition 2. Hunt Institute for Botanical Documentation, Pittsburgh; 2001.* Pittsburgh: Hunt Institute for Botanical Documentation Carnegie Mellon University.

**Davis AJS, Dawson W, Essl F, Kreft H, Lenzner B, Pergl J, Pyšek P, Weigelt P, Winter M, van Kleunen M. 2025.** Global Naturalized Alien Flora (GloNAF). Open access data to support research on understanding global plant invasions. [Data set]. Zenodo. https://doi.org/10.5281/zenodo.14696776

**Denelle P, Weigelt P, Kreft H**. **2023**. GIFT—An R package to access the Global Inventory of Floras and Traits. *Methods in Ecology and Evolution* **14**: 2738–2748.

**Govaerts R**. **2025**. WCVP: World Checklist of Vascular Plants. Facilitated by the Royal Botanic Gardens, Kew. [v14 accessed 28 May 2025

**Govaerts R, Lughadha NE, Black N, Turner R, Paton A**. **2021**. The World Checklist of Vascular Plants, a continuously updated resource for exploring global plant diversity. *Scientific Data* **8**:215.

**Kiss L, Vaghefi N, Bransgrove K, Dearnaley JDW, Takamatsu S, Tan YP, Marston C, Liu SY, Jin DN, Adorada DL, *et al.*** **2020**. Australia: A Continent Without Native Powdery Mildews? *Frontiers in Microbiology* **11**: 1–31.

**Smith SA, Brown JW**. **2018**. Constructing a broadly inclusive seed plant phylogeny. *American Journal of Botany* **105**: 302–314.

**Talhinhas P, Baroncelli R**. **2021**. Colletotrichum species and complexes: geographic distribution, host range and conservation status. *Fungal Diversity* **110**: 109–198.

**Talhinhas P, Baroncelli R**. **2023**. Hosts of Colletotrichum. *Mycosphere* **14**: 158–261.

**Weigelt P, König C, Kreft H. 2020.** GIFT – A Global Inventory of Floras and Traits for macroecology and biogeography. *J Biogeogr* **47**: 16– 43.

**van Kleunen M, Pyšek P, Dawson W, Essl F, Kreft H, Pergl J, Weigelt P, Stein A, Dullinger S, König C, *et al.*** **2019**. The Global Naturalized Alien Flora (GloNAF) database. *Ecology* 100: e02542.

**Weigelt P, Denelle P**. **2022**. GIFT: Access to the Global Inventory of Floras and Traits (GIFT). R package version 1.3.2, https://CRAN.R-project.org/package=GIFT

**Yang Q, Weigelt P, Fristoe TS, Zhang Z, Kreft H, Stein A, Seebens H, Dawson W, Essl F, König C, *et al.*** **2021**. The global loss of floristic uniqueness. *Nature Communications* **12**.
